# *AHDC1* missense mutations in Xia-Gibbs syndrome

**DOI:** 10.1016/j.xhgg.2021.100049

**Published:** 2021-08-10

**Authors:** Michael M. Khayat, Jianhong Hu, Yunyun Jiang, He Li, Varuna Chander, Moez Dawood, Adam W. Hansen, Shoudong Li, Jennifer Friedman, Laura Cross, Emilia K. Bijlsma, Claudia A.L. Ruivenkamp, Francis H. Sansbury, Jeffrey W. Innis, Jessica Omark O’Shea, Qingchang Meng, Jill A. Rosenfeld, Kirsty McWalter, Michael F. Wangler, James R. Lupski, Jennifer E. Posey, David Murdock, Richard A. Gibbs

**Affiliations:** 1 Human Genome Sequencing Center, Baylor College of Medicine, Houston, TX, USA; 2 Department of Molecular and Human Genetics, Baylor College of Medicine, Houston, TX, USA; 3 Medical Scientist Training Program, Baylor College of Medicine, Houston, TX, USA; 4 UCSD Departments of Neuroscience and Pediatrics, Rady Children’s Hospital Division of Neurology, Rady Children’s Institute for Genomic Medicine, San Diego, CA, USA; 5 Department of Pediatrics and Genetics, Children’s Mercy Hospitals, Kansas City, MO, USA; 6 Department of Clinical Genetics, Leiden University Medical Center, Leiden, the Netherlands; 7 All Wales Medical Genomics Service, NHS Wales Cardiff and Vale University Health Board, Institute of Medical Genetics, University Hospital of Wales, Cardiff, UK; 8 Departments of Human Genetics, Pediatrics, and Internal Medicine, University of Michigan, Ann Arbor, MI, USA; 9 Department of Pediatrics, University of Michigan, Ann Arbor, MI, USA; 10 GeneDx, Gaithersburg, MD, USA; 11 Texas Children’s Neurological Research Institute, Houston, TX, USA; 12 Texas Children’s Hospital, Houston, TX, USA; 13 Department of Pediatrics, Baylor College of Medicine, Houston, TX, USA; 14 These authors contributed equally to this work

## Abstract

Xia-Gibbs syndrome (XGS; MIM: 615829) is a phenotypically heterogeneous neurodevelopmental disorder (NDD) caused by newly arising mutations in the AT-Hook DNA-Binding Motif-Containing 1 (*AHDC1*) gene that are predicted to lead to truncated AHDC1 protein synthesis. More than 270 individuals have been diagnosed with XGS worldwide. Despite the absence of an independent assay for AHDC1 protein function to corroborate potential functional consequences of rare variant genetic findings, there are also reports of individuals with XGS-like trait manifestations who have *de novo* missense *AHDC1* mutations and who have been provided a molecular diagnosis of the disorder. To investigate a potential contribution of missense mutations to XGS, we mapped the missense mutations from 10 such individuals to the AHDC1 conserved protein domain structure and detailed the observed phenotypes. Five newly identified individuals were ascertained from a local XGS Registry, and an additional five were taken from external reports or databases, including one publication. Where clinical data were available, individuals with missense mutations all displayed phenotypes consistent with those observed in individuals with *AHDC1* truncating mutations, including delayed motor milestones, intellectual disability (ID), hypotonia, and speech delay. A subset of the 10 reported missense mutations cluster in two regions of the AHDC1 protein with known conserved domains, likely representing functional motifs. Variants outside the clustered regions score lower for computational prediction of their likely damaging effects. Overall, *de novo* missense variants in *AHDC1* are likely diagnostic of XGS when *in silico* analysis of their position relative to conserved regions is considered together with disease trait manifestations.

## Introduction

*De novo* stop-gain and frameshift mutations in the gene encoding the AT-Hook DNA-Binding Motif-Containing 1 (*AHDC1*) protein that are predicted by conceptual translation to lead to truncated AHDC1 protein synthesis are well-established as an underlying cause of Xia-Gibbs syndrome (XGS; MIM: 615829).^1-14^ Reported truncating mutations span most of the length of the protein and include some sites of recurrent, independently arising *de novo* events. AHDC1 likely has a function in the nucleus mediated by its AT-hook binding motifs that are associated with DNA binding.^1,15,16^ Following the identification of the first four XGS cases,^12^ more than 270 individuals with XGS have been identified throughout the world by the XGS family support group and staff at the Baylor College of Medicine (BCM) Human Genome Sequencing Center (HGSC). Eighty-four of these individuals have provided consent for further research and detailed phenotype and *AHDC1* mutation information, which is housed in a dedicated and secure XGS Registry.^8^

As clinical manifestations of XGS overlap with the multitude of other heterogeneous individually rare NDD traits, all diagnoses so far have been dependent on molecular diagnostic testing by DNA sequencing approaches, and the disease is essentially defined by the molecular diagnostic determination of a pathogenic or likely pathogenic variant identified in *AHDC1*.^12^ In the majority of cases, *de novo*, pathogenic *AHDC1* mutations were identified via trio exome sequencing, while plausible variants in other genes were not identified or were excluded based upon absent genotype-phenotype correlation.^4,8,12^ In instances where *de novo* mutation status could not be determined due to the lack of trio-based sequencing data or the lack of DNA samples from both biological parents for segregation studies, the pathogenicity of a truncating *AHDC1* variant was established based on the similarity of the clinical manifestations to other individuals with XGS, coupled with predicted damaging effects of the truncating variants.

Compared to *AHDC1* truncating mutations, it remains challenging to determine which amino acid changes may be deleterious for AHDC1 function. This challenge is further exacerbated by lack of a “biomarker” or laboratory assay to assess protein function. AHDC1 is well conserved across most vertebrates, with 94% identity between human and mouse proteins. The gene is overall intolerant to missense variation, with a positive missense *Z* score of 2.86 and a missense observed-versus-expected mutation ratio of 0.75 reported in the Genome Aggregation Database (gnomAD v.2.1.1).^17^ There are many known rare and ultrarare *AHDC1* variants in the gnomAD population control cohort, however, including 528 missense variants, of which 98% (518) have a minor allele frequency (MAF) < 0.001. It is not known how many individuals who harbor rare variant *AHDC1* alleles as reported in gnomAD may potentially have a mild NDD. Therefore, neither the specific amino acid change nor the allelic frequency of a missense variant is sufficient to infer pathogenicity.

To date, a total of five putatively pathogenic missense variants in *AHDC1* have been reported in the literature or in accessible public databases (Table 1). Each report leveraged the observation of *de novo* occurrence of an *AHDC1* mutation and phenotypic similarity of a new clinical case to the previously reported XGS cases to assert as evidence supportive of pathogenicity. Three of five were in the DECPIHER database, and one was shared via a genetic testing provider. Gumus^6^ described a Turkish individual with a *de novo* mutation leading to an Asp-to-Gly change at amino acid position 1,457 and concluded that this led to craniosynostosis, a new phenotypic feature not previously found in individuals with XGS. Interestingly, an individual in a cohort with craniosynostosis was reported with an *AHDC1 de novo* frameshift mutation (p.C791fs*57).^18^ This is a position with identical recurring *de novo* frameshift mutations in at least five other XGS individuals with no reported craniosynostosis,^1^ and whether this is a phenotypic expansion of the XGS trait or potentially represents a clinical manifestation due to a dual molecular diagnosis and multilocus pathogenic variation (MPV) remains a question.^19^

Three entries in DECIPHER^20^ indicate *de novo* mutations at positions 548, 549, and 1,348 (Gly548Ser, Arg549His, and Ser1348Pro) that have been ascribed to XGS. One variant reported by GeneDx indicates a possible XGS diagnosis for an individual with a *de novo* change at position 487 (Arg487Trp). While the *de novo* origin of these missense variants and shared phenotypes between these individuals and the previously reported XGS clinical spectrum are strongly suggestive of XGS molecular diagnoses, there is no independent functional testing method to show the impact of these changes on molecular function or cellular phenotype to objectively and independently corroborate the findings by an orthogonal experimental functional assay. In some cases, it is not clear which criteria were used to eliminate other possible variants in the genome as potential factors contributing to disease. Therefore, the assignment of each of these *AHDC1* mutations as the underlying cause of the clinical manifestations of these individuals may be premature.

In this study, we report an additional five individuals with missense mutations in *AHDC1* who were provided a molecular and clinical diagnosis of XGS. The genotypic profiles from these individuals, together with the five from earlier reports of missense variants in *AHDC1*, are analyzed (total distinct missense alleles studied: n = 10). This allelic series is the largest and only such study of the *AHDC1* locus. Moreover, we report the objective quantitative analysis of XGS trait manifestations in comparison to well-established pathogenic *AHDC1* truncating variant alleles and to other Mendelizing disorders. Collectively, these analyses provide additional evidence for pathogenicity for some, but not all, of the missense variants in *AHDC1* that have been ascribed to XGS.

## Subjects and methods

### Ethics and consent

Approvals for data use for this study fell into three categories. First, the five individuals who joined the XGS Registry consented for participation under approval by the BCM Institutional Review Board (IRB), protocol number H-39945. Second, data from four individuals were used according to the DECIPHER allowable use agreement or were from published information.^6^ Third, one family provided data as approved by protocol IRB #170447 (Genomic Sequencing in Neurologic Disorders) approved by the University of California at San Diego IRB and Rady Children’s Hospital Research Compliance. As a result, the mutation data for all 10 individuals were available. Partial phenotype data were also available for the five “external” individuals, and detailed clinical data were available for the five individuals who had consented to participation in this study via the XGS Registry.

### Subject recruitment and data security

Affected individuals were initially recruited through social media, e-mail, physician contact, or by word of mouth. The XGS Registry was configured in a RedCap environment,^21^ hosted in a local Health Insurance Portability and Accountability Act (HIPAA)-compliant server. Following initial contact, parents of probands were queried for participation in the XGS Registry and presented with initial consent forms. Next, they were invited to fully consent and to either directly deposit clinical records or to enable their healthcare provider to share their history. Genetic reports and clinical reports were then independently reviewed by BCM HGSC investigators. Additional included individuals (not enrolled in the XGS Registry) were identified through Genematcher^22^ and DECIPHER.^20^

### DNA sequence analysis

The initial molecular diagnoses were by a variety of next-generation DNA sequencing methods (Table 1; Supplemental notes). Follow-up Sanger dideoxy DNA sequencing was performed whenever patient samples were available.

### Subject phenotype assessment

Five individuals from the XGS Registry (Table 1) with available medical reports were reviewed, and clinically ascertained phenotypes were compared to the previously published XGS spectrum.^1,8^ Affected individuals with a report of low muscle tone or hypotonia were indicated under one phenotypic category (“hypotonia”) summarizing the phenotype. Additional phenotypic features that were not part of the previously reported XGS spectrum were also noted. Limited phenotype data were available for three of the five individuals who did not join the XGS Registry, where caregivers provided information (Table 2).

### Computational clustering of phenotypic features

We compared Human Phenotype Ontology (HPO) terms representing the phenotypes of both individuals with XGS due to protein-truncating mutations (n = 34) and the five individuals from the XGS Registry with missense variants to data from Online Mendelian Inheritance in Man (OMIM). The HPO descriptions for OMIM diseases with at least five HPO terms were obtained from the Jackson Laboratory HPO database.^23^ XGS individual phenotypes were converted to HPO terms manually. A word matrix was constructed with OMIM disease or XGS individuals in rows and HPO terms in the columns (0 = absence; 1 = presence). The OMIM disease/XGS individual similarities were determined using cosine similarity algorithm based on the co-occurrences of HPO terms, normalized by term frequency-inverse document frequency aggregated from all the OMIM diseases (scikit-learn package in Python). This procedure resulted in pairwise phenotypic similarities between all the OMIM diseases and individuals with XGS. Pairwise phenotypic similarity scores ranged from 0 (no match) to 1 (highest possible match) and were plotted into networks using igraph in R. We also trimmed the OMIM disease node to keep the diseases with at least one neighbor with similarity score > 0.1 (n = 3,464).

### Computational prediction of functional impact

*AHDC1* missense variants were analyzed by multiple *in silico* pathogenicity prediction algorithms. These methods included Missense Tolerance Ratio (MTR),^24^ Combined Annotation Dependent Depletion (CADD v.1.6),^25^ Functional Analysis through Hidden Markov Models (FATHMM-XF),^26^ and REVEL.^27^ These scores were then compared to those calculated for *AHDC1* missense variants reported in the Genome Aggregation Database (gnomAD v.2.1.1) control cohort. All variants in this study were scored using American College of Medical Genetics and Genomics (ACMG) criteria utilizing VarSome.^28^

## Results

### Mutation profile of putative pathogenic missense *AHDC1* variant alleles

**Figure 1.**
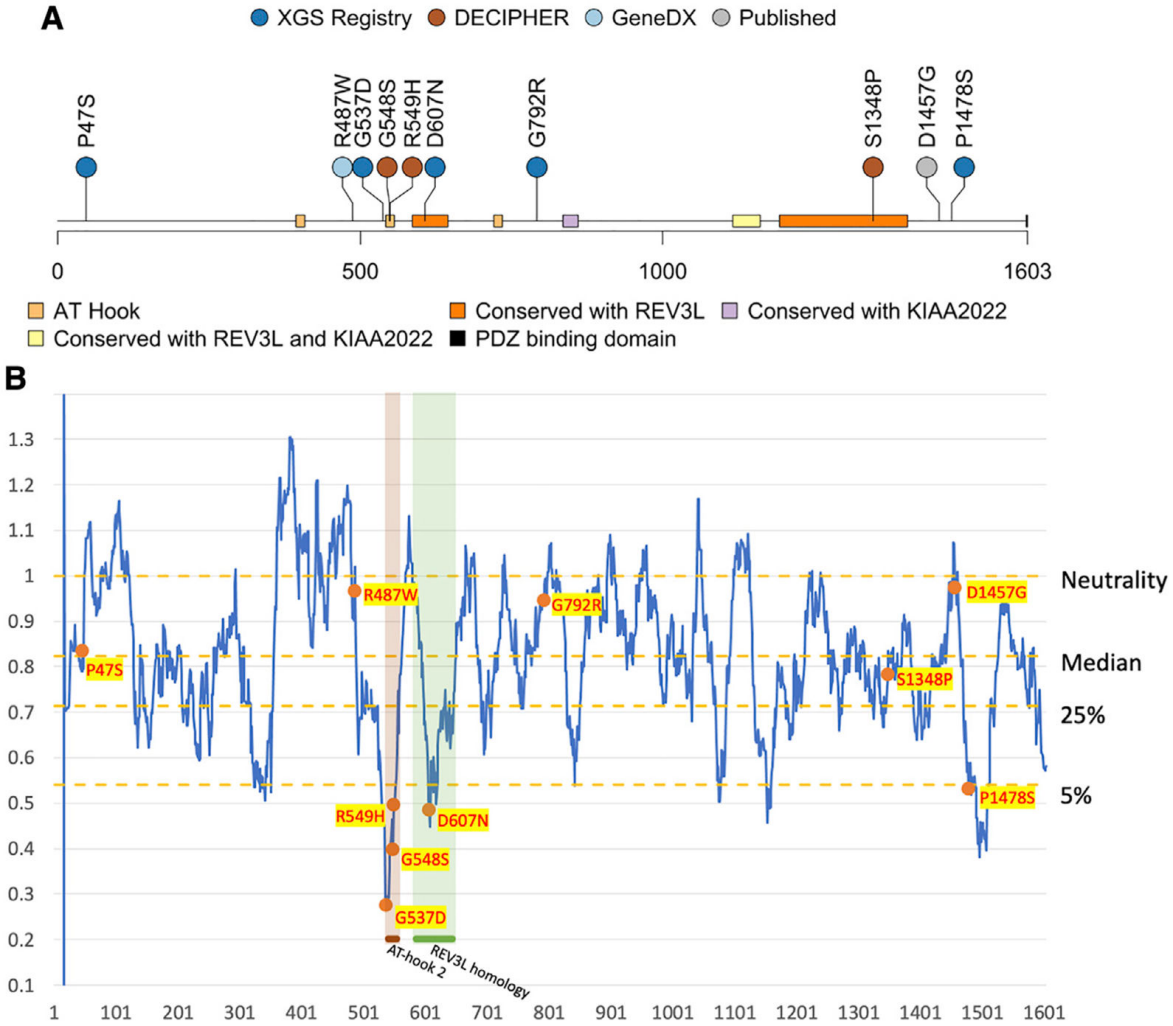
Recorded AHDC1 missense cases and protein sequence mutability (A) A total of 10 individuals with *de novo* or suspected de novo missense mutations in *AHDC1* are shown. (B) The *AHDC1* missense mutations are scored using the missense tolerance ratio score. A lower score indicates a higher intolerance to missense mutations based on sequence conservation of population controls from gnomAD.

A total of 10 individuals with *AHDC1* missense mutations were studied. Five of those individuals were from external reports, and a further five individuals with missense variants in *AHDC1* were separately enrolled in the XGS Registry (Table 1), together with their genetic and clinical details. Based on guidelines from the ACMG, two of the five missense mutations in the XGS Registry were initially classified as likely pathogenic (LP), two were variants of uncertain significance (VUS), and one was classified as likely benign (LB) (Table S1). Among them, four of the five missense variant alleles were confirmed to be *de novo* mutations based on trio sequencing. The *de novo* status for variant p.Pro1478Ser could not be determined, as paternal data were not available. The details of the mutations in these five individuals in the XGS Registry, together with the details of five previously reported missense variant alleles, are shown in Figure 1A and in Table 1. Additional clinical synopsis details are delineated in the individual case reports in the Supplemental notes.

### Clustering of missense variants in *AHDC1* domains

The distribution of the 10 studied putatively pathogenic missense mutations were mapped along the length of the 1,603 amino acid primary sequence of the AHDC1 protein. Of note, two apparent clusters were observed, which included seven of the 10 missense variants. Cluster 1 contained four variants, spanning just 71 amino acid positions (537–607) within or flanking the region of the highly conserved AT-hook domain 2 and cluster 2, a conserved REV3L domain (Domain of Unknown Function 4683 [DUF4683]) (individuals 3–6). Cluster 2 consisted of three variants that spanned 131 residues near the C terminus of the protein, within or near a second domain that is conserved with REV3L (individuals 8–10) (Figure 1). One of these three variants (individual 9) is the mutation in the previously published report of the affected individual of Gumus.^6^ Individual 10 bore a variant in close proximity, for which *de novo* status could not be inferred to provide supportive evidence due to the absence of paternal DNA. The two cluster regions are predicted to be intolerant to missense variation due to purifying selection (Figure 1B; Figure S1).

Three of the 10 missense variants fell outside the clusters. A variant at amino acid position 487 was within 51 residues of the first cluster, but where it “sits” in three-dimensional protein space and secondary and tertiary protein structure is unknown. The variants within individuals 1 (p.Pro47Ser) and 7 (p.Gly792Arg) did not cluster with other variants and the map to undefined AHDC1 protein regions, with no homology to other proteins.

### Computational prediction of pathogenicity

Nine of 10 *de novo* or suspected *de novo* missense mutations in *AHDC1* considered here were predicted as LP using *in silico* pathogenicity scores including CADD and FATHMM-XF (Figure S1), with only the variant in individual 1 showing lower effect. However, variants within the two clusters described above tended to be within the top 10% of the highest pathogenicity score group. In contrast, the variants in the three individuals who were located outside the clusters consistently ranked lower in the calculated overall pathogenicity scores. Scoring using the REVEL meta predictor elevated the missense variants in the first cluster to the top 10%, compared to gnomAD controls.

### Other mutation data

Other data, including variants in genes other than *AHDC1*, were considered as potential contributors to the clinical profiles of the individuals with missense *AHDC1* mutations who were in the XGS Registry. Individual 1, with a *de novo AHDC1* p.Pro47Ser variant, also harbored LP *de novo* missense variant c.10151A>G (p.Asp3384Gly) in FAT Atypical Cadherin 3 (*FAT3*). Although *FAT3* has not been definitively associated with a Mendelian disorder, recently a *FAT3* variant has been implicated as a potential contributor to autism spectrum disorder (ASD).^29^ Further, the *AHDC1 de novo* missense mutation in individual 1 was classified as LB according to ACMG criteria. This classification was supported by the observation of different alleles at the same amino acid position in two individuals in the gnomAD database—although those alleles were not observed in the gnomAD reported “normal” (control) cohort and may also have had disease association.

Individual 2, with missense variant c.1459C>T (p.Arg487Trp), was potentially highly informative for this study, as the mutation occurred at position 487, which was near the proximal boundary of the cluster of four variants spanning amino acid positions 537–607. Consultation with the individual’s caregivers revealed, however, that the initial genetic evaluation of the *AHDC1* missense mutation noted mosaicism, although further details were unavailable. Further, features that were atypical for XGS were noted (Supplemental notes). Overall, it was determined that the p.Arg487Trp change in this individual is not likely to be contributing to the phenotype, although it is possible that in other, non-mosaic individuals the variant may contribute to disease. If the mutation were not pathogenic, then the remaining four changes in the cluster region would span just 71 amino acids, including the critical AT-hook motif.

The diagnosis of individual 8, who harbored a COOH-terminal missense variant allele in *AHDC1* (p.Ser1348Pro) was confounded by the presence of a hemizygous LP *de novo* missense mutation in the HECT, UBA, and WWE Domain Containing E3 Ubiquitin Protein Ligase 1 (*HUWE1* c.9070G>A [p.Ala3024Thr]) gene. Missense mutations in *HUWE1* are highly constrained (missense *Z* score = 8.87);both SNV and duplication Copy Number Variation involving *HUWE1* are known causes of X-linked intellectual disability (ID; MIM: 309590).

No other potentially pathogenic variants were identified in other individuals in this study, although a series of variants were reviewed and determined to be VUS (Table 3).

### Phenotypic spectrum of individuals with an *AHDC1* missense mutation

**Figure 2.**
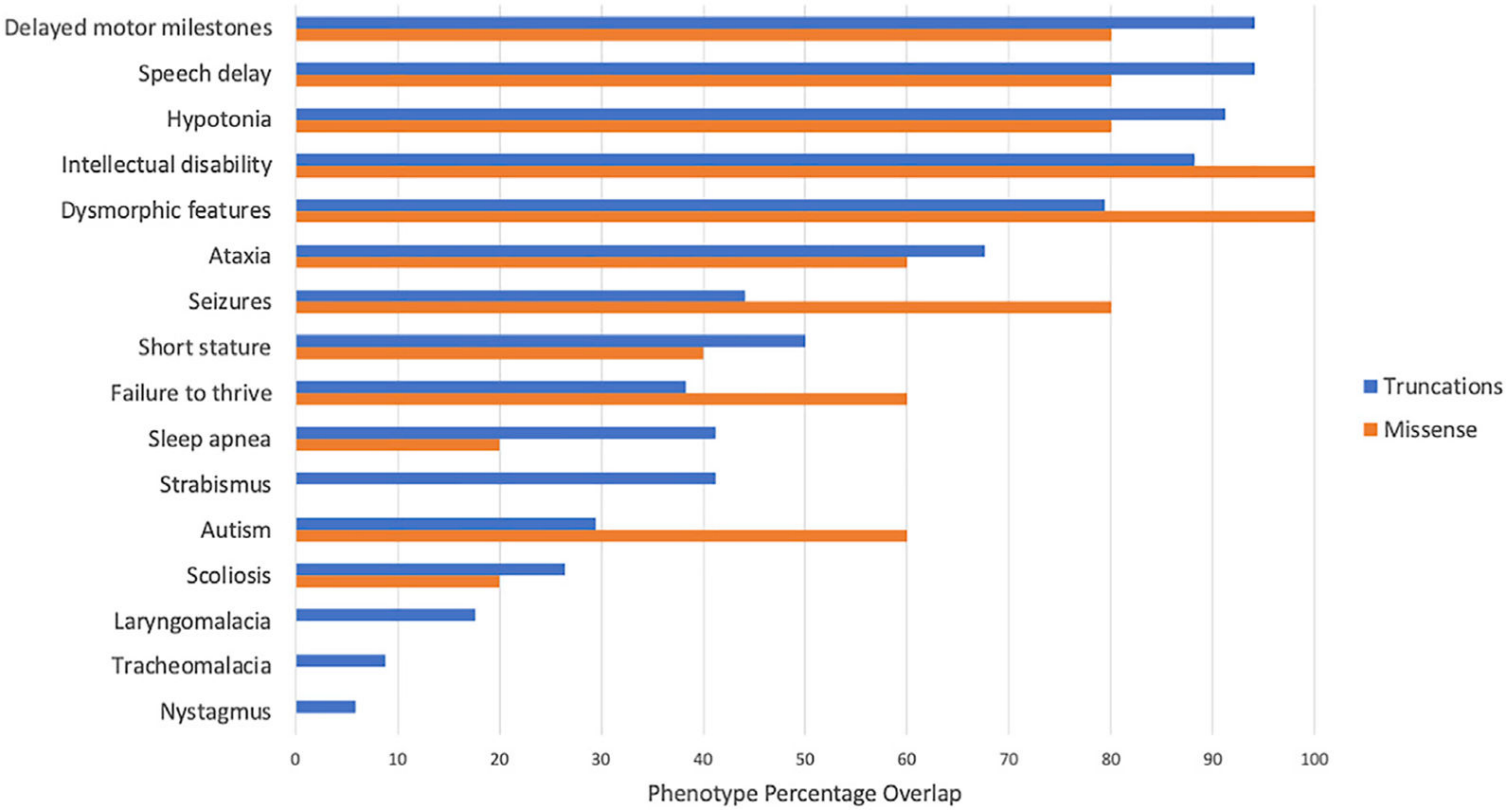
Comparison of XGS phenotypes Data from 34 individuals with XGS due to truncating *AHDC1* mutations were compared with those from 5 individuals with suspected or confirmed *de novo* missense mutations in *AHDC1*, who have joined the XGS Registry.

We previously defined five core and 12 secondary clinically observed phenotypic XGS features, based on the clinical presentations of 34 individuals with XGS due to truncating mutations in *AHDC1*.^1^ The five core phenotypes were observed in >80% of XGS individuals and thus perhaps represent a clinical synopsis of the AD trait associated with this locus. These clinically observed findings include delayed motor milestones, ID, hypotonia, low muscle tone, and speech delay, potentially refining the core *AHDC1*-associated NDD trait. For the current study, we combined hypotonia and low muscle tone into a single category, although the two features were reported separately during most assessments. We compared the occurrence of these core and secondary phenotypes derived from individuals with truncating mutations to those with *AHDC1* missense mutations. These comparisons provided evidence that the spectrum of clinical manifestations of individuals reported with putatively pathogenic missense mutations in *AHDC1* largely overlapped with those harboring truncating mutations (Figure 2).

Of note, all five individuals from the XGS Registry with missense variants had ID, and four of the five exhibited delayed motor milestones, speech delay, and a diagnosis of hypotonia/low muscle tone. Individuals in this study who were ascertained through public databases or previous studies did not have the same consistent phenotypic assessment information available as the information content available for those from the XGS Registry. Phenotypes for subject 2 were not available, and the DECIPHER individuals had limited phenotypic information upon query (Supplemental notes). However, the majority of cases had indicated phenotypes of ID and speech delay with the occurrence of other core phenotypes. An exception to the phenotypic spectrum associated with the missense mutations, compared to that of the truncating mutations, was that four of the five individuals in the XGS Registry with missense mutations reported seizures, compared to an incidence of approximately 50% in the truncation cases. As this feature is of high clinical relevance, we also examined the partial phenotype data available from three of the five external individuals not included in the XGS Registry. Overall, six of eight XGS individuals reported seizures, and data for one were not available (Table 2). Notably, data from individual 10, who had joined the XGS Registry and indicated “no” for this feature, initially indicated that the parents had suspected mild seizures. Subsequent physician records, however, indicated a series of normal EEGs and a record of no seizures. Overall, these data suggest a higher incidence of seizures in this group of individuals with missense *AHDC1* mutations, relative to truncation cases. It is not clear if this might represent a gain-of-function (GoF) versus loss-of-function (LoF) mutational effect.

### Phenotypic clustering of individuals with missense mutations

**Figure 3.**
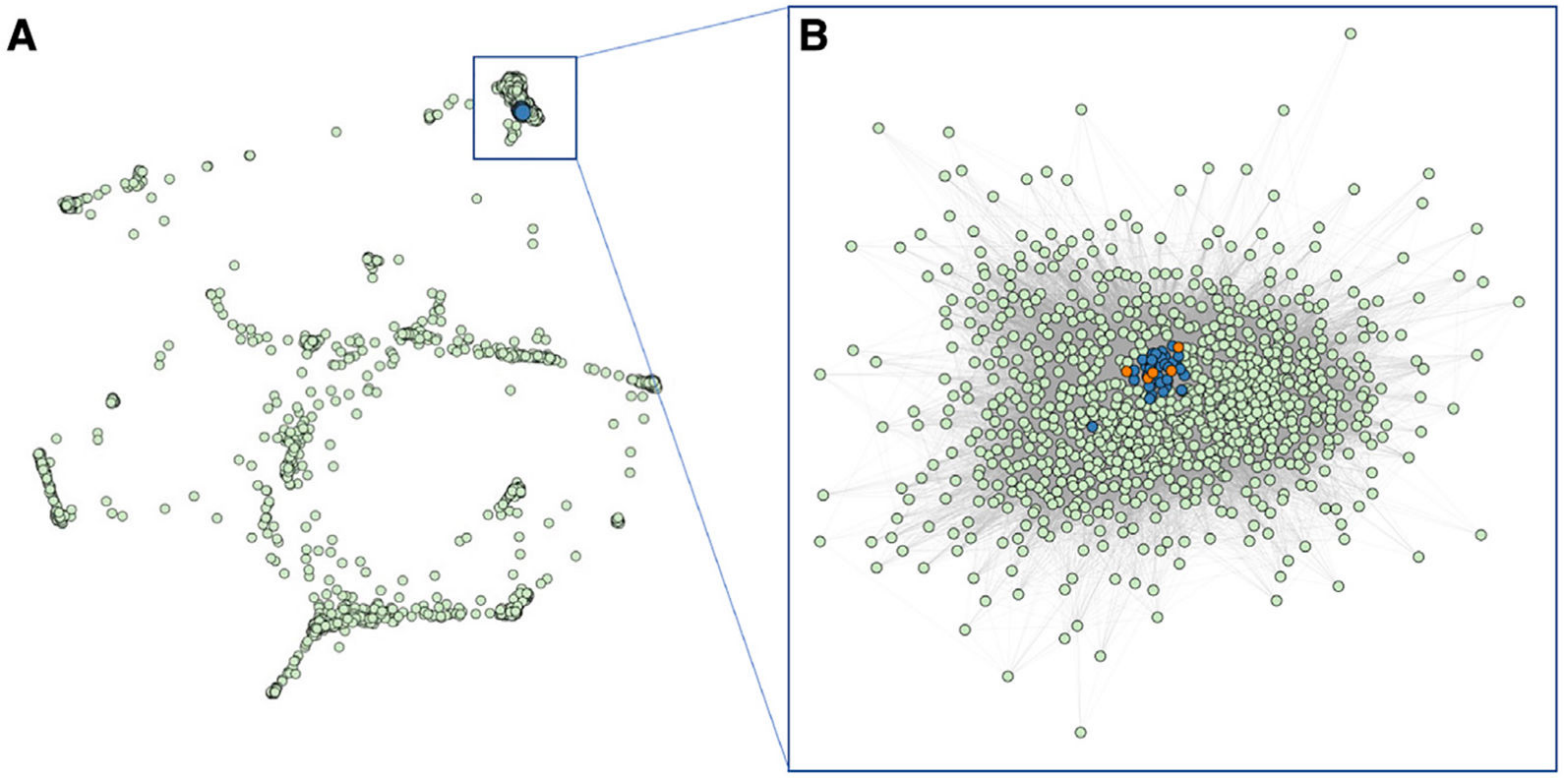
Phenotypic similarity network between individuals with *AHDC1* variants and OMIM diseases (A) Clustering of individuals with an *AHDC1* missense mutation or truncation mutation with 3,464 diseases reported to OMIM based on phenotype similarity illustrated by orange dots, blue dots, and green dots, respectively. (B) Reclustered OMIM disease nodes with at least one connection and similarity >0.1 to individuals with an *AHDC1* missense or truncation mutation.

To further investigate phenotypic similarities between individuals with a *de novo* missense versus a truncation mutation in *AHDC1*, a comparative analysis utilizing the 17 phenotypic terms collected from the XGS Registry was implemented. The 17 observed clinical phenotype features were converted into HPO terms and used to compare the phenotypic similarities among all the individuals with XGS, as well as the clinical manifestations of 3,464 human disease traits as defined by the clinical synopsis of individual entries from OMIM. Data from individuals with missense variants in *AHDC1* clustered together with those from individuals harboring truncating mutations (Figure 3A). In addition, individuals with a truncation or missense mutation were more similar to each other than to other diseases with similar phenotypes (Figure 3B). Collectively, by quantitative phenotyping and objective similarity searches, individuals with missense variants were phenotypically more similar to XGS due to truncating mutations, than to other disorders.

## Discussion

The first 20 individuals reported with an XGS diagnosis bore *de novo* protein-truncating mutations, presumably LoF alleles, leading to speculation that *AHDC1* missense variants may not cause disease.^8^ Alternative interpretations could be that missense variant alleles might potentially cause GoF versus LoF effects, or perhaps a different distinct rare disease trait. Truncating *AHDC1* mutations are also predominant among the more than 270 families with XGS now known worldwide, including 84 who have joined the XGS Registry. Nevertheless, there are at least 10 individuals who have been assigned an XGS diagnosis on the basis that they have *de novo* or suspected *de novo* missense mutations in *AHDC1*. Five of these 10 individuals with missense mutations have joined the XGS Registry and provided detailed clinical phenotyping records for further study and analyses. One additional family provided limited clinical data, and information from the four remaining individuals was drawn from DECIPHER or publication. No biomarker or other laboratory assays are yet available to directly evaluate the biological effect of the amino acid substitutions on AHDC1 protein function, and therefore these molecular diagnoses rely considerably on the *de novo* status of the changes and the absence of other variants/loci that parsimoniously explain the observed clinical features. Further consideration of the genetic and phenotypic data for these reported cases affords the opportunity to substantiate that missense mutations can cause XGS. As also suggested by our XGS data analysis and the *FAT3* variation (individual 1), correlation with clinical phenotyping and expected observations may be useful in molecular diagnostic interpretation, and the presence of multilocus pathogenic variation needs to be considered in the molecular differential.^30^

The amino acid positions of the 10 missense *AHDC1* variants reported as underlying XGS suggest a clustering of events in two primary sequence regions of the protein. One cluster was striking, as it contained 4/10 mutations within 71 amino acid residues—less than 5% of the protein. This cluster also contained an AT-hook binding domain, with two of the missense mutations in the core AT-hook 12-amino-acid motif.^12^ The second, more broadly defined cluster contained three missense variants and spanned 131 amino acid residues—about 8% of the protein. The three remaining mutations did not map to any defined cluster or associate with regions of strong conservation or computationally defined domains. Although the structure of AHDC1 is not solved, and 3D protein effects cannot yet be considered, these data suggest that the variants that arise in either of the two clusters are potentially more likely to perturb normal protein function.

Algorithmic prediction of the likelihood of the missense mutations being damaging to the AHDC1 protein also distinguished variants within the two clusters from the mutations that occurred in surrounding regions. While all 10 of the missense *AHDC1* variants in this study passed the likelihood threshold for deleteriousness using the CADD scores (21.5 to 28.6) to predict their effects, those outside of the clusters had generally lower scores (21.5 to 24.8). FATHMM-XF analysis further differentiated the two classes of variants, with all mutations in the clusters reported as LP and the three additional variants with the lowest scores for pathogenicity (Figure S1C). We also observed the general trend of most of the clustered mutations being in regions of very low MTR scores, indicating high intolerance to change (Figure 1B). Overall, the data and the computational *in silico* analyses support the general model of at least two mutation-sensitive regions within the AHDC1 protein.

The information content of quantitative phenotyping and comparison of phenotypes from individuals with *AHDC1* mutations did not initially provide additional insight into the true impact of the missense mutations. The same overall pattern was observed in individuals with missense mutations versus those with truncating mutations, and when HPO terms were analyzed both groups had essentially the same distance from other neurological conditions in OMIM. This was an expected result, as one of the criteria for the assignment of an XGS diagnosis was having a similar phenotype to the already-reported individuals. The important question of whether there are individuals who harbor missense *AHDC1* alleles with lower functional impact than any of the missense mutations reported here is unanswered. Such individuals may have much milder phenotypes and may not present for clinical evaluation. Hypomorphic alleles may also lead to disease traits with incomplete penetrance, leading to under-ascertainment of individuals with inherited variant alleles. Nevertheless, the quantitative analyses of the XGS Registry data did reveal the association of reported seizures with these missense cases, and that finding was distinguishable from what has been reported for truncating alleles. We speculate that these findings might implicate some potential GoF effects of *AHDC1* missense variants.

Together, the data here suggest guidelines for consideration of the possible pathogenicity of newly observed missense variants in *AHDC1* in individuals with XGS-like phenotypes and in the absence of other genomic variants that explain an individual’s clinical presentation. Foremost, established guidelines from the ACMG should be used to guide variant interpretation. These consider most factors that are contained in the current discussion, including inheritance pattern and computational predictions of likelihood of damaging effects. The *de novo* status of any *AHDC1* missense variant should be considered as a highly important factor for diagnosis, as there is as yet no evidence for any transmitted variant-causing disease. Moreover, the position of the mutation might be considered, with variant alleles mapping within either of the two cluster regions prioritized for assignment as disease causing. There are many caveats to these guidelines, including the imprecision of the knowledge of the cluster boundaries and the likelihood that with improved understanding of AHDC1 structure and function some missense changes outside the clusters may be determined to be pathogenic. Nevertheless, it is clear from both the population data that include many variants in reportedly “normal” individuals and the different presentation of the variant data from individuals 1, 2, and 7 in this study that pathogenicity cannot be confidently asserted from *de novo* status alone. Further, the study underscores the high priority for both accrual of larger datasets and development of independent functional assays for the protein. One further potential insight from our missense clinical data regarding missense constraint and gnomAD-facilitated variant interpretation, and as has also been gleaned from studies of premature truncating codon (PTC) interpretation, there may be limitations to gnomAD-assisted variant interpretation when potentially dealing with GoF mutation effects.^31-33^

The mechanism by which *AHDC1* mutations result in XGS is unknown, and there have been prior suggestions of both haploinsufficiency and dominant negative effects.^1,34^ Missense mutations are expected to result in full-length protein products and are therefore more likely to act in dominant negative (antimorphic) or other GoF (neomorphic and hypermorphic) manners. Consequently, the report here and from prior missense mutations supports the dominant negative model, but it does not exclude that both haploinsufficiency (LoF) and dominant negative (GoF) mechanisms could be at play, depending on the primary mutation.

There are a growing number of early-onset severe neurological Mendelian diseases for which, like XGS, a known gene with *de novo* truncating mutations leads to disease.^35-37^ There is, however, a paucity of well-studied examples where such loci are also damaged by missense variants and yield related phenotypes. This is in part likely due to the complexity of determining causation for missense variants in disease diagnosis. Where previous work has identified pathogenic missense variants in genes that are also associated with autosomal dominant disorders (e.g., *NOTCH3*), the enrichment of missense variants in conserved protein domains has been noted.^38^ This trend is consistent with observations here of missense mutations clustering within and near the conserved AT-hook 2 domain and the REV3L homology domain (DUF4683) in *AHDC1*.

In summary, we report quantitative phenotyping and analyses in 10 *AHDC1* missense mutations—five novel putative XGS molecular diagnoses and five reported mutations. These novel missense *AHDC1* variants newly described in this study provide confirmatory supportive evidence that some missense variants in *AHDC1* can cause XGS. Quantitative clinical phenotyping reveals missense alleles share a core monoallelic NDD autosomal dominant trait phenotype with that observed in association with LoF variant alleles but suggests that seizures may more likely be observed in association with missense alleles. To what extent such findings might implicate potential GoF effects remains to be determined by more extensive studies of *AHDC1* allelic series.

## Supplementary Material

1

## Figures and Tables

**Table 1.** Individuals with an identified *de novo* or suspected *de novo* missense mutation in *AHDC1*

Individual #	Nucleotide change	Protein change	Data type	Source
1	c.139C>T	p.Pro47Ser	exome sequencing	XGS Registry
2	c.1459C>T	p.Arg487Trp	exome sequencing	GeneDx
3	c.1610G>A	p.Gly537Asp	comprehensive NGS panel; microarray	XGS Registry
4	c.1642G>A	p.Gly548Ser	WGS/targeted sequencing	DECIPHER (#287553)
5	c.1646G>A	p.Arg549His	exome sequencing; SNP array	DECIPHER (#370261)
6	c.1819G>A	p.Asp607Asn	exome sequencing	XGS Registry
7	c.2374G>C	p.Gly792Arg	exome sequencing; CGH array	XGS Registry, GeneDx
8	c.4042T>C	p.Ser1348Pro	exome sequencing	DECIPHER (#277992)
9	c.4370A>G	p.Asp1457Gly	exome sequencing	PMID 30858058
10^a^	c.4432C>T	p.Pro1478Ser	exome sequencing	XGS Registry

Individuals who joined the XGS Registry also contributed clinical data for this study. The source of data for the other individuals is indicated. Other genetic tests that were also administered are noted under the data type. NGS, next-generation sequencing; WGS, whole-genome sequencing; CGH, comparative genomic hybridization.

a Suspected *de novo* mutation.

**Table 2.** Phenotypes, genotypes, and demographic features of individuals with an *AHDC1* missense mutation

Patient ID	1	3	5	6	7	8	9	10
**Mutation**								
Nucleotide change	c.139C>T	c.1610G>A	c.1646G>A	c.1819G>A	c.2374G>C	c.4042T>C	c. 4370A>G	c.4432C>T
Protein change	p.Pro47Ser	p.Gly537Asp	p.Arg549His	p.Asp607Asn	p.Gly792Arg	p.Ser1348Pro	p.Asp1457Gly	p.Pro1478Ser
Age	14 years	10 years	6 years	23 years	12 years	10 years	2 years	11 years
Sex	M	F	F	M	F	M	F	F
Ethnicity	white	African American/white	white	white	white	white	NA	Latino/Hispanic
**Growth**								
Stature (percentile)	<10th	99th	>90th	43rd	99th	30th	1st	1st
Scoliosis	Y	N	N	N	N	N	NA	N
**Comprehensive skills and language**								
M-CHAT score	4	NA	NA	15	4		NA	0
Autism diagnosis	Y	N	N	Y	N	Y	NA	Y
Current language^a^	3	3	2	3	3	0	1	1
Age at first word	11 months	3 years	~2 years	2.5 years	2 years	NA	NA	2–3 years
Age using two words together	~2 years		~4 years	~12–13 years	not recalled		NA	NA
Age at following command	2 years		NA	NA	has trouble following command	not reported	NA	1.5 years
**Mobility**								
Hypotonia diagnosis	Y	N	N	Y	Y	Y	Y	Y
Independent walking	Y	Y	Y	Y	walking with support	Y		Y
Age at independent walking	~2 years	11 months	15 months	1.5 years		2 years		1 year
**Sleep/airway**								
Sleep apnea	N	Y	N	N	N			N
Using breathing support	N	Y (CPAP at night)	N	N	N			N
**Neurology**								
MRI	normal	NA	not done	abnormal	abnormal	abnormal	abnormal	abnormal
EEG	normal	NA	NA	NA	normal	NA	abnormal	normal
Seizure	Y	Y	NA	Y	Y	Y	Y	N
Age at first seizure	3 years	NA		22 years	2–3 years	6 years	3 days	NA
Ataxia	N	N	N	Y	Y			Y
**Vision**								
Wearing glasses or contacts	N	Y	N	N	Y	Y	N	N
Visual acuity	20/30	hyperopia, night blindness	normal	NA	NA		hypermetropia	hypermetropia	NA
Strabismus	N	N	N	N	N	Y	Y	N
**Dysmorphic features**								
Features	coarse facial features	long palpebral fissures, deep-set eyes, hypertelorism, macrocephaly, cleft palate	broad forehead, thin upper lip	macrocephaly (likely familial)	upslanted palpebral fissures, microcephaly, low-set ears	broad forehead, wide nasal bridge, brachycephaly, microtia, clinodactyly 5th finger, mild microcephaly	almond-shaped eyes, thin upper lip, brachycephaly, microcephaly, protuberant ears	upslanting palpebral fissures, microcephaly

Of the total of 10 individuals, five joined the XGS Registry and provided all available clinical data (individuals 1, 3, 6, 7, and 10). Partial data were available for three of the additional five known individuals (5, 8, 9). M-CHAT, Modified Checklist for Autism in Toddlers; CPAP, continuous positive airway pressure; MRI, magnetic resonance imaging; EEG, electroencephalogram; M, male; F, female; Y, yes; N, no; NA, not applicable.

a Current language: 0, no words; 1, <50 words; 2, no sentence but >50 words; and 3, full sentence >200 words.

**Table 3.** Additional genetic findings in individuals with *AHDC1 de novo* missense mutations

Case #	Gene(s)	Nucleotide change	Amino acid change	Zygosity	Inheritance pattern	gnomAD AC/AF	Predicted pathogenicity
1	*AHDC1*	c.139C>T	p.Pro47Ser	heterozygous	*de novo*	0/0	likely benign
	*FAT3*	c.10151A>G	p.Asp3384Gly	heterozygous	*de novo*	0/0	likely pathogenic
	*SERPINA1*	c.863A>G	p.Glu288Val	heterozygous	maternal	0/0	uncertain significance
3	*AHDC1*	c.1610G>A	p.Gly537Asp	heterozygous	*de novo*	0/0	likely pathogenic
	*ANK3*	c.6715C>T	p.Arg2239Cys	heterozygous	paternal	13/0.00005	uncertain significance
	*APC2*	c.4958G>A	p.Arg1653Gln	heterozygous	paternal	58/0.0004	uncertain significance
	*C5orf42*	c.8397A>C	p.Lys2799Asn	heterozygous	maternal	0/0	uncertain significance
	*SON*	c.313A>G	p.Thr105Ala	heterozygous	paternal	9/0.0004	uncertain significance
	*TTC19*	c.380A>G	p.Tyr127Cys	heterozygous	paternal	0/0	uncertain significance
	*TPM3, C1orf189, C1orf43, UBAP2L, HAX1, MIR190B*	microdeletion within 1q21.3	(154,150,447-154,255,258)x1	heterozygous	unknown	NA	uncertain significance
6	*AHDC1*	c.1819G>A	p.Asp607Asn	heterozygous	*de novo*	0/0	likely pathogenic
	*DNAH14*	c.409C>T	p.Arg137*	heterozygous	paternal	198/0.0007	uncertain significance
	*DNAH14*	c.13548A>T	p.*4516Tyrfs*5	heterozygous	maternal	1,216/0.007	uncertain significance
7	*AHDC1*	c.2374G>C	p.Gly792Arg	heterozygous	*de novo*	0/0	uncertain significance
	*NPHP1*	microdeletion within 2q13	(110,199,004-110,337,690)x1	heterozygous	paternal	NA	uncertain significance
	*ATP11, CXorf661, MIR505*	duplication within Xq27.1	(138,699,164-139,089,567)x3	heterozygous	maternal	NA	uncertain significance
8^a^	*AHDC1*	c.4042T>C	p.Ser1348Pro	heterozygous	*de novo*	0/0	likely pathogenic
	*HUWE1*	c.9070G>A	p.Ala3024Thr	hemizygous	*de novo*	0/0	likely pathogenic
	*NEB*	c.9139C>A	p.His3047Asn	heterozygous	paternal	191/0.0005	benign
	*NEB*	c.7343G>A	p.Arg2448His	heterozygous	maternal	33/0.00008	benign

Data for individuals 1, 3, 6, and 7 were from the XGS Registry. Individual 10, also in the XGS Registry, did not report additional considered variants. AC/AF, allele count/allele frequency.

a Data for individual 8, not in the Registry, were provided with consent by the individual’s health provider. Predicted pathogenicity was assessed using VarSome as described in Subjects and methods.

## References

[1] Khayat MM, Li H, Chander V, Hu J, Hansen AW, Li S, Traynelis J, Shen H, Weissenberger G, Stossi F, (2021). Phenotypic and protein localization heterogeneity associated with AHDC1 pathogenic protein-truncating alleles in Xia–Gibbs syndrome. Hum. Mutat 42, 577–591. 33644933 10.1002/humu.24190 PMC8115934

[2] Cardoso-Dos-Santos AC, Oliveira Silva T, Silveira Faccini A, Woycinck Kowalski T, Bertoli-Avella A, Morales Saute JA, Schuler-Faccini L, and de Oliveira Poswar F (2020). Novel *AHDC1* Gene Mutation in a Brazilian Individual: Implications of Xia-Gibbs Syndrome. Mol. Syndromol 11, 24–29. 32256298 10.1159/000505843 PMC7109424

[3] Cheng X, Tang F, Hu X, Li H, Li M, Fu Y, Yan L, Li Z, Gou P, Su N, (2019). Two Chinese Xia-Gibbs syndrome patients with partial growth hormone deficiency. Mol. Genet. Genomic Med 7, e00596. 30729726 10.1002/mgg3.596 PMC6465669

[4] Díaz-Ordoñez L, Ramirez-Montaño D, Candelo E, Cruz S, and Pachajoa H (2019). Syndromic Intellectual Disability Caused by a Novel Truncating Variant in AHDC1: A Case Report. Iran. J. Med. Sci 44, 257–261. 31182893 PMC6525729

[5] García-Acero M, and Acosta J (2017). Whole-Exome Sequencing Identifies a de novo *AHDC1* Mutation in a Colombian Patient with Xia-Gibbs Syndrome. Mol. Syndromol 8, 308–312. 29230160 10.1159/000479357 PMC5701272

[6] Gumus E (2020). Extending the phenotype of Xia-Gibbs syndrome in a two-year-old patient with craniosynostosis with a novel de novo AHDC1 missense mutation. Eur. J. Med. Genet 63, 103637. 30858058 10.1016/j.ejmg.2019.03.001

[7] He P, Yang Y, Zhen L, and Li D-Z (2020). Recurrent hypoplasia of corpus callosum as a prenatal phenotype of Xia-Gibbs syndrome caused by maternal germline mosaicism of an AHDC1 variant. Eur. J. Obstet. Gynecol. Reprod. Biol 244, 208–210. 31812316 10.1016/j.ejogrb.2019.11.031

[8] Jiang Y, Wangler MF, McGuire AL, Lupski JR, Posey JE, Khayat MM, Murdock DR, Sanchez-Pulido L, Ponting CP, Xia F, (2018). The phenotypic spectrum of Xia-Gibbs syndrome. Am. J. Med. Genet. A 176, 1315–1326. 29696776 10.1002/ajmg.a.38699 PMC6231716

[9] Mubungu G, Makay P, Boujemla B, Yanda S, Posey JE, Lupski JR, Bours V, Lukusa P, Devriendt K, and Lumaka A (2021). Clinical presentation and evolution of Xia-Gibbs syndrome due to p.Gly375ArgfsTer3 variant in a patient from DR Congo (Central Africa). Am. J. Med. Genet. A 185, 990–994. 33372375 10.1002/ajmg.a.62049 PMC9235023

[10] Murdock DR, Jiang Y, Wangler M, Khayat MM, Sabo A, Juusola J, McWalter K, Schatz KS, Gunay-Aygun M, and Gibbs RA (2019). Xia-Gibbs syndrome in adulthood: a case report with insight into the natural history of the condition. Cold Spring Harb. Mol. Case Stud 5, a003608. 30622101 10.1101/mcs.a003608 PMC6549549

[11] Ritter AL, McDougall C, Skraban C, Medne L, Bedoukian EC, Asher SB, Balciuniene J, Campbell CD, Baker SW, Denenberg EH, (2018). Variable Clinical Manifestations of Xia-Gibbs syndrome: Findings of Consecutively Identified Cases at a Single Children’s Hospital. Am. J. Med. Genet. A 176, 1890–1896. 30152016 10.1002/ajmg.a.40380

[12] Xia F, Bainbridge MN, Tan TY, Wangler MF, Scheuerle AE, Zackai EH, Harr MH, Sutton VR, Nalam RL, Zhu W, (2014). De novo truncating mutations in AHDC1 in individuals with syndromic expressive language delay, hypotonia, and sleep apnea. Am. J. Hum. Genet 94, 784–789. 24791903 10.1016/j.ajhg.2014.04.006 PMC4067559

[13] Yang S, Li K, Zhu M-M, Yuan X-D, Jiao X-L, Yang Y-Y, Li J, Li L, Zhang HN, Du YH, (2019). Rare Mutations in AHDC1 in Patients with Obstructive Sleep Apnea. BioMed Res. Int 2019, 5907361. 31737670 10.1155/2019/5907361 PMC6815587

[14] Yang H, Douglas G, Monaghan KG, Retterer K, Cho MT, Escobar LF, Tucker ME, Stoler J, Rodan LH, Stein D, (2015). De novo truncating variants in the AHDC1 gene encoding the AT-hook DNA-binding motif-containing protein 1 are associated with intellectual disability and developmental delay. Cold Spring Harb. Mol. Case Stud 1, a000562. 27148574 10.1101/mcs.a000562 PMC4850891

[15] Thul PJ, Åkesson L, Wiking M, Mahdessian D, Geladaki A, Ait Blal H, Alm T, Asplund A, Björk L, Breckels LM, (2017). A subcellular map of the human proteome. Science 356, eaal3321. 28495876 10.1126/science.aal3321

[16] Uhlén M, Fagerberg L, Hallström BM, Lindskog C, Oksvold P, Mardinoglu A, Sivertsson Å, Kampf C, Sjöstedt E, Asplund A, (2015). Proteomics. Tissue-based map of the human proteome. Science 347, 1260419. 25613900 10.1126/science.1260419

[17] Karczewski KJ, Francioli LC, Tiao G, Cummings BB, Alföldi J, Wang Q, Collins RL, Laricchia KM, Ganna A, Birnbaum DP, ; Genome Aggregation Database Consortium (2020). The mutational constraint spectrum quantified from variation in 141,456 humans. Nature 581, 434–443. 32461654 10.1038/s41586-020-2308-7 PMC7334197

[18] Miller KA, Twigg SRF, McGowan SJ, Phipps JM, Fenwick AL, Johnson D, Wall SA, Noons P, Rees KE, Tidey EA, (2017). Diagnostic value of exome and whole genome sequencing in craniosynostosis. J. Med. Genet 54, 260–268. 27884935 10.1136/jmedgenet-2016-104215 PMC5366069

[19] Karaca E, Posey JE, Coban Akdemir Z, Pehlivan D, Harel T, Jhangiani SN, Bayram Y, Song X, Bahrambeigi V, Yuregir OO, (2018). Phenotypic expansion illuminates multilocus pathogenic variation. Genet. Med 20, 1528–1537. 29790871 10.1038/gim.2018.33 PMC6450542

[20] Firth HV, Richards SM, Bevan AP, Clayton S, Corpas M, Rajan D, Van Vooren S, Moreau Y, Pettett RM, and Carter NP (2009). DECIPHER: Database of Chromosomal Imbalance and Phenotype in Humans Using Ensembl Resources. Am. J. Hum. Genet 84, 524–533. 19344873 10.1016/j.ajhg.2009.03.010 PMC2667985

[21] Harris PA, Taylor R, Minor BL, Elliott V, Fernandez M, O’Neal L, McLeod L, Delacqua G, Delacqua F, Kirby J, Duda SN; and The REDCap consortium (2019). Building an international community of software platform partners. J. Biomed. Inform 95, 103208. 31078660 10.1016/j.jbi.2019.103208 PMC7254481

[22] Sobreira N, Schiettecatte F, Valle D, and Hamosh A (2015). GeneMatcher: a matching tool for connecting investigators with an interest in the same gene. Hum. Mutat 36, 928–930. 26220891 10.1002/humu.22844 PMC4833888

[23] Köhler S, Gargano M, Matentzoglu N, Carmody LC, Lewis-Smith D, Vasilevsky NA, Danis D, Balagura G, Baynam G, Brower AM, (2021). The human phenotype ontology in 2021. Nucleic Acids Res. 49 (D1), D1207–D1217. 33264411 10.1093/nar/gkaa1043 PMC7778952

[24] Traynelis J, Silk M, Wang Q, Berkovic SF, Liu L, Ascher DB, Balding DJ, and Petrovski S (2017). Optimizing genomic medicine in epilepsy through a gene-customized approach to missense variant interpretation. Genome Res. 27, 1715–1729. 28864458 10.1101/gr.226589.117 PMC5630035

[25] Rentzsch P, Witten D, Cooper GM, Shendure J, and Kircher M (2019). CADD: predicting the deleteriousness of variants throughout the human genome. Nucleic Acids Res. 47 (D1), D886–D894. 30371827 10.1093/nar/gky1016 PMC6323892

[26] Rogers MF, Shihab HA, Mort M, Cooper DN, Gaunt TR, and Campbell C (2018). FATHMM-XF: accurate prediction of pathogenic point mutations via extended features. Bioinformatics 34, 511–513. 28968714 10.1093/bioinformatics/btx536 PMC5860356

[27] Ioannidis NM, Rothstein JH, Pejaver V, Middha S, McDonnell SK, Baheti S, Musolf A, Li Q, Holzinger E, Karyadi D, (2016). REVEL: An Ensemble Method for Predicting the Pathogenicity of Rare Missense Variants. Am. J. Hum. Genet 99, 877–885. 27666373 10.1016/j.ajhg.2016.08.016 PMC5065685

[28] Kopanos C, Tsiolkas V, Kouris A, Chapple CE, Albarca Aguilera M, Meyer R, and Massouras A (2019). VarSome: the human genomic variant search engine. Bioinformatics 35, 1978–1980. 30376034 10.1093/bioinformatics/bty897 PMC6546127

[29] Baldwin I, Shafer RL, Hossain WA, Gunewardena S, Veatch OJ, Mosconi MW, and Butler MG (2021). Genomic, clinical, and behavioral characterization of 15q11.2 bp1-bp2 deletion (burnside-butler) syndrome in five families. Int. J. Mol. Sci 22, 1–24. 10.3390/ijms22041660 PMC7914695 33562221

[30] Posey JE, Harel T, Liu P, Rosenfeld JA, James RA, Coban Akdemir ZH, Walkiewicz M, Bi W, Xiao R, Ding Y, (2017). Resolution of Disease Phenotypes Resulting from Multilocus Genomic Variation. N. Engl. J. Med 376, 21–31. 27959697 10.1056/NEJMoa1516767 PMC5335876

[31] Poli MC, Ebstein F, Nicholas SK, de Guzman MM, Forbes LR, Chinn IK, Mace EM, Vogel TP, Carisey AF, Benavides F, ; Undiagnosed Diseases Network members (2018). Heterozygous Truncating Variants in POMP Escape Nonsense-Mediated Decay and Cause a Unique Immune Dysregulatory Syndrome. Am. J. Hum. Genet 102, 1126–1142. 29805043 10.1016/j.ajhg.2018.04.010 PMC5992134

[32] Bayram Y, White JJ, Elcioglu N, Cho MT, Zadeh N, Gedikbasi A, Palanduz S, Ozturk S, Cefle K, Kasapcopur O, ; Baylor-Hopkins Center for Mendelian Genomics (2017). REST Final-Exon-Truncating Mutations Cause Hereditary Gingival Fibromatosis. Am. J. Hum. Genet 101, 149–156. 28686854 10.1016/j.ajhg.2017.06.006 PMC5501868

[33] Coban-Akdemir Z, White JJ, Song X, Jhangiani SN, Fatih JM, Gambin T, Bayram Y, Chinn IK, Karaca E, Punetha J, ; Baylor-Hopkins Center for Mendelian Genomics (2018). Identifying Genes Whose Mutant Transcripts Cause Dominant Disease Traits by Potential Gain-of-Function Alleles. Am. J. Hum. Genet 103, 171–187. 30032986 10.1016/j.ajhg.2018.06.009 PMC6081281

[34] Quintero-Rivera F, Xi QJ, Keppler-Noreuil KM, Lee JH, Higgins AW, Anchan RM, Roberts AE, Seong IS, Fan X, Lage K, (2015). MATR3 disruption in human and mouse associated with bicuspid aortic valve, aortic coarctation and patent ductus arteriosus. Hum. Mol. Genet 24, 2375–2389. 25574029 10.1093/hmg/ddv004 PMC4380077

[35] Yang Y, Muzny DM, Reid JG, Bainbridge MN, Willis A, Ward PA, Braxton A, Beuten J, Xia F, Niu Z, (2013). Clinical whole-exome sequencing for the diagnosis of mendelian disorders. N. Engl. J. Med 369, 1502–1511. 24088041 10.1056/NEJMoa1306555 PMC4211433

[36] Bamshad MJ, Shendure JA, Valle D, Hamosh A, Lupski JR, Gibbs RA, Boerwinkle E, Lifton RP, Gerstein M, Gunel M, ; Centers for Mendelian Genomics (2012). The Centers for Mendelian Genomics: a new large-scale initiative to identify the genes underlying rare Mendelian conditions. Am. J. Med. Genet. A 158A, 1523–1525. 22628075 10.1002/ajmg.a.35470 PMC3702263

[37] Posey JE, O’Donnell-Luria AH, Chong JX, Harel T, Jhangiani SN, Coban Akdemir ZH, Buysek S, Pehlivan D, Carvalho CMB, Baxter S, (2019). Insights into genetics, human biology and disease gleaned from family based genomic studies. Genet. Med 21, 798–812. 30655598 10.1038/s41436-018-0408-7 PMC6691975

[38] Turner TN, Douville C, Kim D, Stenson PD, Cooper DN, Chakravarti A, and Karchin R (2015). Proteins linked to autosomal dominant and autosomal recessive disorders harbor characteristic rare missense mutation distribution patterns. Hum. Mol. Genet 24, 5995–6002. 26246501 10.1093/hmg/ddv309 PMC6354780

