# Supplementary material for: AHDC1 missense mutations in Xia-Gibbs syndrome
Source: HGG Adv. 2021 Aug 10;2(4):100049. doi: 10.1016/j.xhgg.2021.100049 (PMC8694554; doi:10.1016/j.xhgg.2021.100049)
Supplement: Document 1. Figure S1, Table S1, and Supplemental notes [file mmc1.pdf]

HGGA, Volume *2*

Supplemental information

AHDC1 missense mutations in Xia-Gibbs syndrome

Michael M. Khayat, Jianhong Hu, Yunyun Jiang, He Li, Varuna Chander, Moez Dawood, Adam W. Hansen, Shoudong Li, Jennifer Friedman, Laura Cross, Emilia K. Bijlsma, Claudia A.L. Ruivenkamp, Francis H. Sansbury, Jeffrey W. Innis, Jessica Omark O'Shea, Qingchang Meng, Jill A. Rosenfeld, Kirsty McWalter, Michael F. Wangler, James R. Lupski, Jennifer E. Posey, David Murdock, and Richard A. Gibbs

Supplemental Information:

Supplementary Figure 1: Evaluation of the frequency and pathogenicity of missense mutations compared to gnomAD controls. (A) Allele frequency vs protein codon for *AHDC1* missense mutations occurring in gnomAD controls. Analysis of individuals with *de novo* or suspected *de novo* missense mutations using pathogenicity scores from CADD (B), FATHMM-XF (C) and REVEL (D); gnomAD controls are displayed as grey dots and cases as red dots.

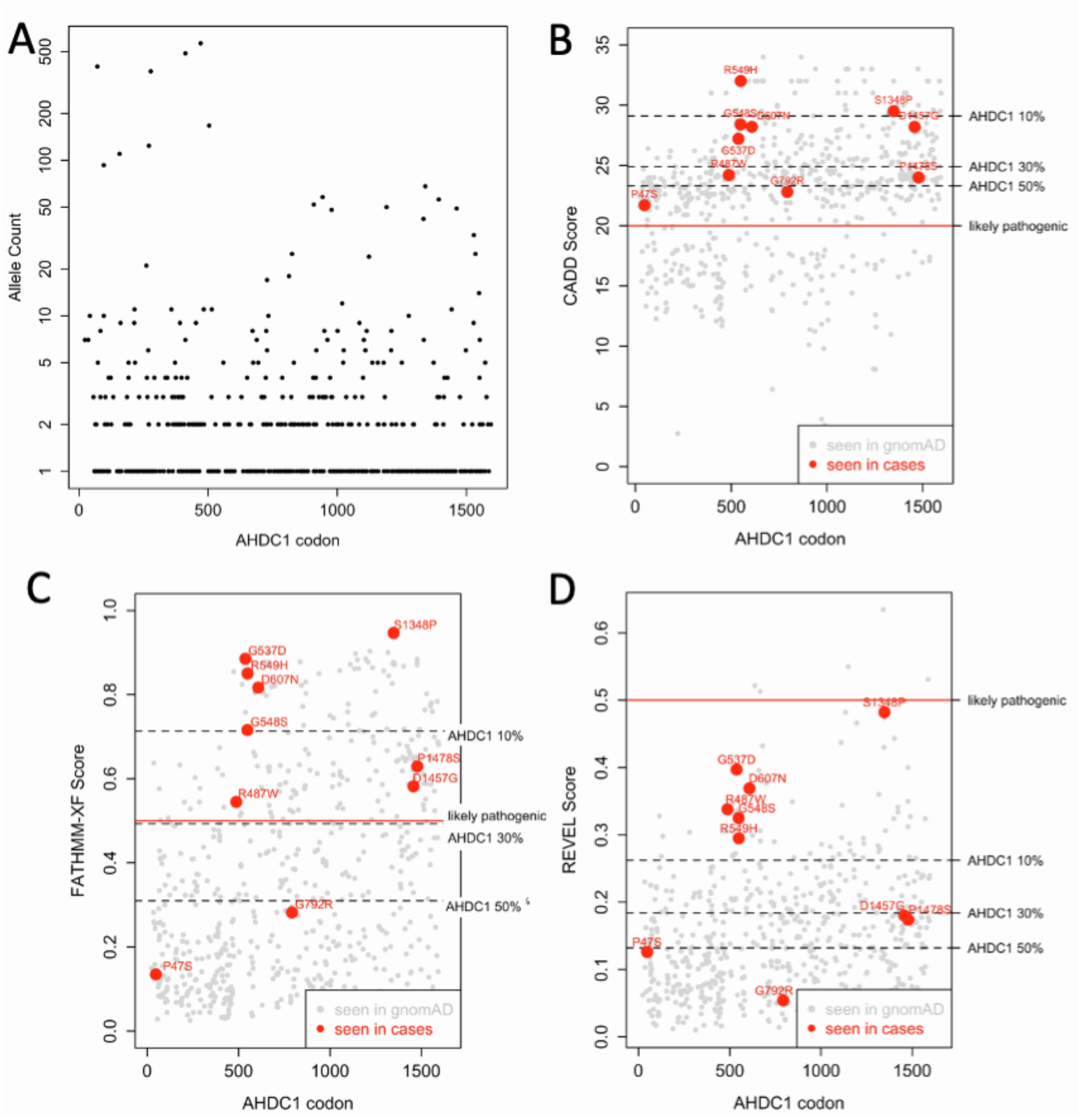

Supplemental Table 1. The missense variants in the *AHDC1* gene were classified using the ACMG standards and guidelines. PS2, PM2 and PP3 are the categories that can be applied to interpret the variants. PS2: *de novo* in a patient with the disease with both maternity and paternity confirmed and no family history, a strong evidence of pathogenicity. PM2: extremely low frequency in general population database gnomAD, a moderate evidence of pathogenicity. PP3: multiple lines of computational evidence support a deleterious effect on the gene or gene product, a supporting evidence of pathogenicity.

**Case #**	**Nucleotide change**	**Protein change**	**ACMG classification**
1	c.139C>T	p.Pro47Ser	Likely Benign
2	c.1459C>T	p.Arg487Trp	Likely Pathogenic
3	c.1610G>A	p.Gly537Asp	Likely Pathogenic
4	c.1642G>A	p.Gly548Ser	Likely Pathogenic
5	c.1646G>A	p.Arg549His	Likely Pathogenic
6	c.1819G>A	p.Asp607Asn	Likely Pathogenic
7	c.2374G>C	p.Gly792Arg	Uncertain Significance
8	c.4042T>C	p.Ser1348Pro	Likely Pathogenic
9	c.4370A>G	p.Asp1457Gly	Likely Pathogenic
10	c.4432C>T	p. Pro1478Ser	Uncertain Significance

Case Reports:

Case 1: is a 14-year-old male with cognitive developmental delay, speech delay, myopathy, coarse facial features, brachydactyly, small hands and feet, history of torticollis and precocious pubarche.

The patient has recurrent respiratory infections since 1 week old. Whole body bone scan by nuclear imaging at 12 years showed that he has skeletal dysplasia, including scoliosis, pectus carinatum, clubbed fingers, thickening of large bones, shortened bilateral femoral neck, enlarged and broad vertebrae, broad long bones, and short segment syrinx. X-ray and MRI showed an enchondroma in the left humerus. He was diagnosed with elevated liver enzymes at 11 years. He also has other chronic phenotypes including seasonal allergies, asthma, acid reflux, constipation and insomnia.

Neurology: The patient had a normal brain magnetic resonance imaging (MRI) at 3 years. He has sensory issues and was diagnosed with dysautonomia at 12 years.

Behavior: He has anxiety and obsessive-compulsive disorder. He has some autistic-like characteristics but was not diagnosed with autism. He has echolalia and will not answer questions without repeating the phrase that is stated to him.

Sequencing studies: Exome sequencing identified a *de novo* c.139C>T (p.Pro47Ser) variant in *AHDC1*. Two other variants in *FAT3* (c.10151A>G, p.Asp3384Gly; *de novo*) and *SERPINA1* (c.10151A>G, p. Glu288Val; maternally inherited) genes were also identified.

Referral: Direct contact by patient family/self-referral. This family consented to participation in the XGS Registry.

Case 2:

This individual has a neurodegenerative phenotype characterized by generalized dystonia and imaging showing basal ganglia mineralization. Consultation with the caregiver and the referring physician identified that while XGS was considered, the atypical clinical phenotype in conjunction with the mosaic nature of the variant ruled out the XGS diagnosis.

Sequencing studies: Exome sequencing identified a mosaic *de novo* c.1459C>T (p.Arg487Trp) variant in *AHDC1*.

Referral: Direct contact by individual's caregiver and referring physician. This family declined full participation in the XGS Registry, however agreed to share the data included in this publication.

Case 3: is a 10-year-old female with bifid uvula, learning delays, obesity, tall stature, and a questionable submucous cleft palate. The patient was born at 35 weeks of gestation via spontaneous vaginal delivery after an unremarkable pregnancy. She never passed newborn hearing screening. She smiled at 4 months of age, rolled over at 3 months of age and first walked at 11 months old. Her first words came at 3 years of age, which was likely helped by the placement of pressure equalization tubes.

The patient is 160.10 cm (> 99.9 %ile) and 114.20 kg (99.99 %ile) with body mass index (BMI) of 44.55 kg/m^2 at 10 years of age.

Neurology: This patient has not been evaluated by neurology, nor has she had a brain MRI.

Behavior: This patient has learning problems and a history of speech delays and food-seeking behavior. She does have behavior problems including aggression.

Sequencing studies:

This patient initially had a microarray, which identified a heterozygous ~105kb deletion within chromosome band 1q21.3 that contains exons 1 and 2 the *TPM3* gene, entire coding regions of *C1orf189*, *C1orf43*, *UBAP2L* and *HAX1* genes. The patient was also negative for Fragile X and Prader-Willi testing. A clinical feature-driven NGS panel testing identified a *de novo AHDC1* c.1610G>A (p.Gly537Asp) mutation in this patient. Other possible candidates are listed in Table 3.

Referral: This family consented to participation in the XGS Registry. Patient phenotype and case report were provided by caregiver.

Case 4: is an 11-year-old male registered in DECIPHER with learning and developmental delays. The patient was reported to have decreased fetal movement. Certain musculoskeletal abnormalities were reported including a positional foot deformity and congenital muscular torticollis.

Neurology: Cognitive impairment, delayed speech and language development and developmental regression.

Sequencing studies: A *de novo* missense variant was reported in *AHDC1* (c.1642G>A, p.Gly548Ser), initially identified by WGS and confirmed by targeted *AHDC1* sequencing.

Referral: Data described are from DECIPHER – consent was obtained directly from the parent for inclusion of this entry in this publication.

Case 5: is a 6-year-old girl, born as the first child of healthy non-consanguineous White parents. Her younger brother is healthy, and the family history is unremarkable. She was born full term after an uncomplicated pregnancy by vacuum-assisted vaginal delivery and weighed 4850 grams. Delivery was complicated by shoulder dystocia, resulting in obstetric brachial plexus injury (Erb's palsy). In the first year she was unable to use her right arm properly, but after extensive physiotherapy her palsy fully recovered.

The patient's motor development was within normal limits. She walked at 15 months. She was in good health, and she had normal hearing and vision. She was referred to the audiologic department at the age of 2y8m for evaluation of speech delay. She spoke her first word around 14 months but hardly had any speech at age 3. Her overall development was delayed. A nonverbal IQ test (SON-R 2-8) at 3 years of age showed that she functioned at a level of 1.5 years. She could talk in short

sentences at the age of 5. She could count and had learned to write numbers. She had just started to learn to read, and she could recognize the alphabet.

At 3 years and 9 months of age her height was 105.5 cm (+0.6 SDS), weight 17.4 kg (+0.3 SDS for height) with head circumference of 51 cm (+0.7 SDS). She has a wide forehead with a metopic ridge and a thin upper lip but is otherwise not dysmorphic. Apart from tapering fingers, she had no abnormalities of hands and feet.

Neurology: The patient did not have a brain MRI.

Behavior: The patient has no behavioral problems.

Sequencing studies: This patient was first seen by a clinical geneticist at age 3 years 9 months. SNP array (CytoScan HD Array Affymetrix) showed 2 copy number variants that were interpreted as LB variants. She has a small interstitial deletion of chromosome 4q of at least 261.2 kb (200 probes) from 98,349,035 bp to 98,610,262 bp (Hg19/GRCh37). The deletion includes one coding gene (*STPG2*), which is not linked to human disease. Furthermore, she has a small interstitial duplication of chromosome 12q of at least 358.5 kb (240 probes), from 123,656,808 bp to 124,015,352 bp. The duplication includes 8 coding genes (*MPHOSPH9*, *CDK2AP1*, *C12orf65*, *SBNO1*, *RILPL2*, *SETD8*, *SNRNP35*, and *RILPL1*). Karyotype: 46, XY; arr[GRCh37] 4q22.3(98349035_98610262)x1, 12q24.31(123656808_124015352)x3. Exome sequencing identified a *de novo AHDC1* c.1646G>A (p.Arg549His) mutation.

Referral: This case was included in DECIPHER database. Patient caregiver provided patient phenotype and case report.

Case 6: is a 23-year-old male with autism spectrum disorder, speech delay and intellectual disability. The major clinical features of this individual are listed in Table 2. He was born at full term with unremarkable pregnancy history. He was referred to a child development clinic at the age of 15 months for not walking or standing independently. He started walking without support at 18 months. He had his first word at approximately 2.5 years of age and did not start using two-word sentences until 12-13yo. He has severe impairment of receptive language.

Patient 6 is of average stature. He has a prominent sacral dimple.

Neurology: The patient had multiple MRIs performed. The first one was performed at the age of 14 months due to suspicion of macrocephaly. Some mild enlargement in the ventricles and extra-axial fluid spaces were noted but did not really appear to be clinically significant. The maternal family was also noted to have mild familial macrocephaly.

Behavior: The family of this patient reported some aggressive behavior starting at the age of 11, including mainly self-injurious behavior (striking his head and temple, etc.) and some physical aggression towards the family. The frequency and duration significantly decreased once he reached 14 years of age (1-2 per year, lasted no more than a minute compared to 5-10 minutes before).

Sequencing studies: Exome and WGS independently identified a *de novo* c.1819G>A (p.Asp607Asn) mutation in *AHDC1*. The mutation is also absent in his four unaffected siblings. Other possible candidates are listed in Table 3.

Referral: This family consented to participation in the XGS Registry.

Case 7: is a 12-year-old female with relative microcephaly, developmental delay, tight heel cords, significant behavior problems and sleep disruption. The pregnancy was complicated by a diagnosis of Lyme disease during the second trimester. This patient was delivered by emergency C-section at 42 weeks of gestation due to breech position. She had feeding difficulty for the first six months and had gastroesophageal reflux requiring treatment in the first year. She sat at 10-11 months and walked at 14-15 months. She spoke her first word beyond 2 years of age. At present, she is using full sentences with a vocabulary of more than 200 words, but she has trouble with receptive language. The patient is of tall stature, 82% in height at 8yo.

Renal and bladder MRI at the age of 9 showed that she has a small cyst in her left kidney and she has a small and thick-walled bladder.

Neurology: Brain MRI at 3 years showed molar tooth sign. Brain MRI at 10yo showed craniofacial and brain malformations, compatible with genetic encephalopathy and Joubert syndrome-related disorder. This patient had a full-scale IQ in the range of 68 and 72.

Behavior: This patient had long-standing behavioral issues since she was a toddler. She has been on multiple medications for her behavior since 3-4yo. She has long-standing ADHD in addition to depression and anxiety and has been diagnosed with disruptive mood disorder. Her family reported worse behavior since approximately age 9 with some puberty changes. Her behavior seems to worsen on approximately a monthly cycle, but she has yet started menstruation. She was hospitalized at age 10 years due to suicidal and homicidal ideation.

Sequencing studies: This patient was initially tested by chromosomal microarray, and a deletion of 2q13 and a duplication of region Xq27.1 were identified. The Xq27.1 duplication was also found in her mother. The 2q13 deletion, which encompasses the *NPHP1* gene that is associated with autosomal recessive Joubert syndrome, appeared to be paternally inherited per exome sequencing. Exome sequencing identified a *de novo* c.2374G>C(p.Gly792Arg) mutation in *AHDC1* and was classified as a variant of uncertain significance; no additional variants in NPHP1 were identified by ES.

Referral: This family consented to participate in the XGS Registry.

Case 8: is a 10-year-old male registered in the DECIPHER with autism and global developmental delay. Dysmorphic features included esotropia, hypermetropia, microtia, brachycephaly, a broad forehead, horizontal eyebrows, a wide nasal bridge and 5th finger clinodactyly. The patient suffered from recurrent respiratory infections and had an intussusception as an infant.

Neurology: Global developmental delay, absent speech at 6 years, and autism spectrum disorder. MRI head at 11 months showed ventriculomegaly and decreased white matter. Occipitofrontal circumference measurements at 2 and 6 years showed postnatal microcephaly.

Sequencing studies: through the DDD project, a *de novo* missense variant was reported in *AHDC1* (c.4042T>C, p.Ser1348Pro) and a *de novo* hemizygous mutation was reported in *HUWE1* (c.9070G>A, p.Ala3024Thr). He is also compound heterozygous for two *NEB* variants (c.9139C>A, p.His3047Asn, paternal and c.7343G>A, p.Arg2448His, maternal). Previous ClinVar classifications of these variants are as variants of uncertain clinical significance and as LB. There are no reported features of a nemaline myopathy. They also had a normal karyotype and array-CGH (comparative genome hybridization),

Referral: Phenotype and genotype are from the DECIPHER database and a clinical notes review.

Case 9: Refer to Gumus (2020).

Case 10: is a 11-year-old female with global developmental delay, microcephaly, gait abnormalities, and speech apraxia. There has been a major concern with recurrent falls, one of which led to a fractured elbow that required surgery. She is unable to run. She wears braces on her feet. She has difficulty with sleep onset for which she takes melatonin. She has been evaluated in sleep medicine and has had a normal sleep study. An iron deficiency was identified for which she takes supplemental iron. She has deficient upper right premolars. Her ears are normally positioned and well-formed. Eyes are spaced appropriately. Eyebrows are normal. Palpebral fissures are

slightly upslanting. Irides are intact. Nose is normal. Philtrum and lips are normal. Jaw is well formed. She has a history of conductive hearing loss. Her mother also reports that she has joint and muscle pain. She has a 6-year-old sibling who is apparently normal.

There were no teratogenic exposures or illnesses during pregnancy and no neonatal problems. Development was delayed, and hearing loss has been an issue that responded to PE tubes and was secondary to numerous infections. She has no history of hospitalizations. Her immunizations are up to date. At present time, she may use approximately 10 single words but does not use word combinations. She exhibits relatively good comprehension. She is able to express her wants and needs using a tablet computer and a picture-based system. There has been no interval regression of any of her language skills.

Neurology: Brain MRI completed at 8 years identified dysgenesis of the corpus callosum and deep white matter T2 hyperintensity. She has had no seizures.

Behavior: She has behavioral concerns including tantrums. In school, she receives autism services, but it is primarily language that is her problem. She likes to socialize. She likes to communicate as well as she can, and there is evident eye contact. Her mother also explained that she was felt to have a lot of words until she was about 2 years of age and then lost the use of some of these.

Sequencing studies: Exome sequencing identified a VUS in *AHDC1* (c.4432C>T, p.P1478S). The variant was not inherited from the mother, but it is unclear if the father has the same variant. An additional heterozygous variant in *UBE3B* was detected that is known to cause Kaufman

oculocerebrofacial syndrome in an autosomal recessive fashion; hence, it is unlikely to explain the observed clinical features.

Referral: This family consented to participate in the XGS Registry.
