# Supplementary material for: AHDC1 missense mutations in Xia-Gibbs syndrome
Source: HGG Adv. 2021 Aug 10;2(4):100049. doi: 10.1016/j.xhgg.2021.100049 (PMC8694554; doi:10.1016/j.xhgg.2021.100049)
Supplement: Document 2. Article plus supplemental information [file mmc2.pdf]

HGG Advances

ARTICLE

AHDC1 missense mutations in Xia-Gibbs syndrome

Michael M. Khayat,[1,2,14] Jianhong Hu,[1,14] Yunyun Jiang,[1,14] He Li,[1] Varuna Chander,[1,2] Moez Dawood,[1,2,3] Adam W. Hansen,[1,2] Shoudong Li,[1] Jennifer Friedman,[4] Laura Cross,[5] Emilia K. Bijlsma,[6] Claudia A.L. Ruivenkamp,[6] Francis H. Sansbury,[7] Jeffrey W. Innis,[8] Jessica Omark O'Shea,[9] Qingchang Meng,[1] Jill A. Rosenfeld,[2] Kirsty McWalter,[10] Michael F. Wangler,[2,11] James R. Lupski,[1,2,12,13] Jennifer E. Posey,[2] David Murdock,[1,2] and Richard A. Gibbs[1,2,*]

Summary

Xia-Gibbs syndrome (XGS; MIM: 615829) is a phenotypically heterogeneous neurodevelopmental disorder (NDD) caused by newly arising mutations in the AT-Hook DNA-Binding Motif-Containing 1 (*AHDC1*) gene that are predicted to lead to truncated AHDC1 protein synthesis. More than 270 individuals have been diagnosed with XGS worldwide. Despite the absence of an independent assay for AHDC1 protein function to corroborate potential functional consequences of rare variant genetic findings, there are also reports of individuals with XGS-like trait manifestations who have *de novo* missense *AHDC1* mutations and who have been provided a molecular diagnosis of the disorder. To investigate a potential contribution of missense mutations to XGS, we mapped the missense mutations from 10 such individuals to the AHDC1 conserved protein domain structure and detailed the observed phenotypes. Five newly identified individuals were ascertained from a local XGS Registry, and an additional five were taken from external reports or databases, including one publication. Where clinical data were available, individuals with missense mutations all displayed phenotypes consistent with those observed in individuals with *AHDC1* truncating mutations, including delayed motor milestones, intellectual disability (ID), hypotonia, and speech delay. A subset of the 10 reported missense mutations cluster in two regions of the AHDC1 protein with known conserved domains, likely representing functional motifs. Variants outside the clustered regions score lower for computational prediction of their likely damaging effects. Overall, *de novo* missense variants in *AHDC1* are likely diagnostic of XGS when *in silico* analysis of their position relative to conserved regions is considered together with disease trait manifestations.

Introduction

De novo stop-gain and frameshift mutations in the gene encoding the AT-Hook DNA-Binding Motif-Containing 1 (*AHDC1*) protein that are predicted by conceptual translation to lead to truncated AHDC1 protein synthesis are well-established as an underlying cause of Xia-Gibbs syndrome (XGS; MIM: 615829).[1–14] Reported truncating mutations span most of the length of the protein and include some sites of recurrent, independently arising *de novo* events. AHDC1 likely has a function in the nucleus mediated by its AT-hook binding motifs that are associated with DNA binding.[1,15,16] Following the identification of the first four XGS cases,[12] more than 270 individuals with XGS have been identified throughout the world by the XGS family support group and staff at the Baylor College of Medicine (BCM) Human Genome Sequencing Center (HGSC). Eighty-four of these individuals have provided consent for further research and detailed phenotype and *AHDC1* mutation information, which is housed in a dedicated and secure XGS Registry.[8]

As clinical manifestations of XGS overlap with the multitude of other heterogeneous individually rare NDD traits, all diagnoses so far have been dependent on molecular diagnostic testing by DNA sequencing approaches, and the disease is essentially defined by the molecular diagnostic determination of a pathogenic or likely pathogenic variant identified in *AHDC1*.[12] In the majority of cases, *de novo*, pathogenic *AHDC1* mutations were identified via trio exome sequencing, while plausible variants in other genes were not identified or were excluded based upon absent genotype-phenotype correlation.[4,8,12] In instances where *de novo* mutation status could not be determined due to the lack of trio-based sequencing data or the lack of DNA samples from both biological parents for segregation studies, the pathogenicity of a truncating *AHDC1* variant was established based on the similarity of the clinical manifestations to other individuals with XGS, coupled with predicted damaging effects of the truncating variants.

[1]Human Genome Sequencing Center, Baylor College of Medicine, Houston, TX, USA; [2]Department of Molecular and Human Genetics, Baylor College of Medicine, Houston, TX, USA; [3]Medical Scientist Training Program, Baylor College of Medicine, Houston, TX, USA; [4]UCSD Departments of Neuroscience and Pediatrics, Rady Children's Hospital Division of Neurology, Rady Children's Institute for Genomic Medicine, San Diego, CA, USA; [5]Department of Pediatrics and Genetics, Children's Mercy Hospitals, Kansas City, MO, USA; [6]Department of Clinical Genetics, Leiden University Medical Center, Leiden, the Netherlands; [7]All Wales Medical Genomics Service, NHS Wales Cardiff and Vale University Health Board, Institute of Medical Genetics, University Hospital of Wales, Cardiff, UK; [8]Departments of Human Genetics, Pediatrics, and Internal Medicine, University of Michigan, Ann Arbor, MI, USA; [9]Department of Pediatrics, University of Michigan, Ann Arbor, MI, USA; [10]GeneDx, Gaithersburg, MD, USA; [11]Texas Children's Neurological Research Institute, Houston, TX, USA; [12]Texas Children's Hospital, Houston, TX, USA; [13]Department of Pediatrics, Baylor College of Medicine, Houston, TX, USA
[14]These authors contributed equally to this work
*Correspondence: agibbs@bcm.edu
https://doi.org/10.1016/j.xhgg.2021.100049.
© 2021 The Authors. This is an open access article under the CC BY license (http://creativecommons.org/licenses/by/4.0/).

Table 1. Individuals with an identified *de novo* or suspected *de novo* missense mutation in *AHDC1*

Individual #	Nucleotide change	Protein change	Data type	Source
1	c.139C>T	p.Pro47Ser	exome sequencing	XGS Registry
2	c.1459C>T	p.Arg487Trp	exome sequencing	GeneDx
3	c.1610G>A	p.Gly537Asp	comprehensive NGS panel; microarray	XGS Registry
4	c.1642G>A	p.Gly548Ser	WGS/targeted sequencing	DECIPHER (#287553)
5	c.1646G>A	p.Arg549His	exome sequencing; SNP array	DECIPHER (#370261)
6	c.1819G>A	p.Asp607Asn	exome sequencing	XGS Registry
7	c.2374G>C	p.Gly792Arg	exome sequencing; CGH array	XGS Registry, GeneDx
8	c.4042T>C	p.Ser1348Pro	exome sequencing	DECIPHER (#277992)
9	c.4370A>G	p.Asp1457Gly	exome sequencing	PMID 30858058
10[a]	c.4432C>T	p.Pro1478Ser	exome sequencing	XGS Registry

Individuals who joined the XGS Registry also contributed clinical data for this study. The source of data for the other individuals is indicated. Other genetic tests that were also administered are noted under the data type. NGS, next-generation sequencing; WGS, whole-genome sequencing; CGH, comparative genomic hybridization.
[a]Suspected *de novo* mutation.

Compared to *AHDC1* truncating mutations, it remains challenging to determine which amino acid changes may be deleterious for AHDC1 function. This challenge is further exacerbated by lack of a "biomarker" or laboratory assay to assess protein function. AHDC1 is well conserved across most vertebrates, with 94% identity between human and mouse proteins. The gene is overall intolerant to missense variation, with a positive missense *Z* score of 2.86 and a missense observed-versus-expected mutation ratio of 0.75 reported in the Genome Aggregation Database (gnomAD v.2.1.1).[17] There are many known rare and ultra-rare *AHDC1* variants in the gnomAD population control cohort, however, including 528 missense variants, of which 98% (518) have a minor allele frequency (MAF) < 0.001. It is not known how many individuals who harbor rare variant *AHDC1* alleles as reported in gnomAD may potentially have a mild NDD. Therefore, neither the specific amino acid change nor the allelic frequency of a missense variant is sufficient to infer pathogenicity.

To date, a total of five putatively pathogenic missense variants in *AHDC1* have been reported in the literature or in accessible public databases (Table 1). Each report leveraged the observation of *de novo* occurrence of an *AHDC1* mutation and phenotypic similarity of a new clinical case to the previously reported XGS cases to assert as evidence supportive of pathogenicity. Three of five were in the DECPIHER database, and one was shared via a genetic testing provider. Gumus[6] described a Turkish individual with a *de novo* mutation leading to an Asp-to-Gly change at amino acid position 1,457 and concluded that this led to craniosynostosis, a new phenotypic feature not previously found in individuals with XGS. Interestingly, an individual in a cohort with craniosynostosis was reported with an *AHDC1 de novo* frameshift mutation (p.C791fs*57).[18] This is a position with identical recurring *de novo* frameshift mutations in at least five other XGS individuals with no reported craniosynostosis,[1] and whether this is a phenotypic expansion of the XGS trait or potentially represents a clinical manifestation due to a dual molecular diagnosis and multilocus pathogenic variation (MPV) remains a question.[19]

Three entries in DECIPHER[20] indicate *de novo* mutations at positions 548, 549, and 1,348 (Gly548Ser, Arg549His, and Ser1348Pro) that have been ascribed to XGS. One variant reported by GeneDx indicates a possible XGS diagnosis for an individual with a *de novo* change at position 487 (Arg487Trp). While the *de novo* origin of these missense variants and shared phenotypes between these individuals and the previously reported XGS clinical spectrum are strongly suggestive of XGS molecular diagnoses, there is no independent functional testing method to show the impact of these changes on molecular function or cellular phenotype to objectively and independently corroborate the findings by an orthogonal experimental functional assay. In some cases, it is not clear which criteria were used to eliminate other possible variants in the genome as potential factors contributing to disease. Therefore, the assignment of each of these *AHDC1* mutations as the underlying cause of the clinical manifestations of these individuals may be premature.

In this study, we report an additional five individuals with missense mutations in *AHDC1* who were provided a molecular and clinical diagnosis of XGS. The genotypic profiles from these individuals, together with the five from earlier reports of missense variants in *AHDC1*, are analyzed (total distinct missense alleles studied: $n = 10$). This allelic series is the largest and only such study of the *AHDC1* locus. Moreover, we report the objective quantitative analysis of XGS trait manifestations in comparison to well-established pathogenic *AHDC1* truncating variant alleles and to other Mendelizing disorders. Collectively, these analyses provide additional evidence for

pathogenicity for some, but not all, of the missense variants in *AHDC1* that have been ascribed to XGS.

Subjects and methods

Ethics and consent

Approvals for data use for this study fell into three categories. First, the five individuals who joined the XGS Registry consented for participation under approval by the BCM Institutional Review Board (IRB), protocol number H-39945. Second, data from four individuals were used according to the DECIPHER allowable use agreement or were from published information.[6] Third, one family provided data as approved by protocol IRB #170447 (Genomic Sequencing in Neurologic Disorders) approved by the University of California at San Diego IRB and Rady Children's Hospital Research Compliance. As a result, the mutation data for all 10 individuals were available. Partial phenotype data were also available for the five "external" individuals, and detailed clinical data were available for the five individuals who had consented to participation in this study via the XGS Registry.

Subject recruitment and data security

Affected individuals were initially recruited through social media, e-mail, physician contact, or by word of mouth. The XGS Registry was configured in a RedCap environment,[21] hosted in a local Health Insurance Portability and Accountability Act (HIPAA)-compliant server. Following initial contact, parents of probands were queried for participation in the XGS Registry and presented with initial consent forms. Next, they were invited to fully consent and to either directly deposit clinical records or to enable their healthcare provider to share their history. Genetic reports and clinical reports were then independently reviewed by BCM HGSC investigators. Additional included individuals (not enrolled in the XGS Registry) were identified through Genematcher[22] and DECIPHER.[20]

DNA sequence analysis

The initial molecular diagnoses were by a variety of next-generation DNA sequencing methods (Table 1; Supplemental notes). Follow-up Sanger dideoxy DNA sequencing was performed whenever patient samples were available.

Subject phenotype assessment

Five individuals from the XGS Registry (Table 1) with available medical reports were reviewed, and clinically ascertained phenotypes were compared to the previously published XGS spectrum.[1,8] Affected individuals with a report of low muscle tone or hypotonia were indicated under one phenotypic category ("hypotonia") summarizing the phenotype. Additional phenotypic features that were not part of the previously reported XGS spectrum were also noted. Limited phenotype data were available for three of the five individuals who did not join the XGS Registry, where caregivers provided information (Table 2).

Computational clustering of phenotypic features

We compared Human Phenotype Ontology (HPO) terms representing the phenotypes of both individuals with XGS due to protein-truncating mutations ($n = 34$) and the five individuals from the XGS Registry with missense variants to data from Online Mendelian Inheritance in Man (OMIM). The HPO descriptions for OMIM diseases with at least five HPO terms were obtained from the Jackson Laboratory HPO database.[23] XGS individual phenotypes were converted to HPO terms manually. A word matrix was constructed with OMIM disease or XGS individuals in rows and HPO terms in the columns ($0 =$ absence; $1 =$ presence). The OMIM disease/XGS individual similarities were determined using cosine similarity algorithm based on the co-occurrences of HPO terms, normalized by term frequency-inverse document frequency aggregated from all the OMIM diseases (scikit-learn package in Python). This procedure resulted in pairwise phenotypic similarities between all the OMIM diseases and individuals with XGS. Pairwise phenotypic similarity scores ranged from 0 (no match) to 1 (highest possible match) and were plotted into networks using igraph in R. We also trimmed the OMIM disease node to keep the diseases with at least one neighbor with similarity score > 0.1 ($n = 3{,}464$).

Computational prediction of functional impact

AHDC1 missense variants were analyzed by multiple *in silico* pathogenicity prediction algorithms. These methods included Missense Tolerance Ratio (MTR),[24] Combined Annotation Dependent Depletion (CADD v.1.6),[25] Functional Analysis through Hidden Markov Models (FATHMM-XF),[26] and REVEL.[27] These scores were then compared to those calculated for *AHDC1* missense variants reported in the Genome Aggregation Database (gnomAD v.2.1.1) control cohort. All variants in this study were scored using American College of Medical Genetics and Genomics (ACMG) criteria utilizing VarSome.[28]

Results

Mutation profile of putative pathogenic missense *AHDC1* variant alleles

A total of 10 individuals with *AHDC1* missense mutations were studied. Five of those individuals were from external reports, and a further five individuals with missense variants in *AHDC1* were separately enrolled in the XGS Registry (Table 1), together with their genetic and clinical details. Based on guidelines from the ACMG, two of the five missense mutations in the XGS Registry were initially classified as likely pathogenic (LP), two were variants of uncertain significance (VUS), and one was classified as likely benign (LB) (Table S1). Among them, four of the five missense variant alleles were confirmed to be *de novo* mutations based on trio sequencing. The *de novo* status for variant p.Pro1478Ser could not be determined, as paternal data were not available. The details of the mutations in these five individuals in the XGS Registry, together with the details of five previously reported missense variant alleles, are shown in Figure 1A and in Table 1. Additional clinical synopsis details are delineated in the individual case reports in the Supplemental notes.

Clustering of missense variants in *AHDC1* domains

The distribution of the 10 studied putatively pathogenic missense mutations were mapped along the length of the 1,603 amino acid primary sequence of the AHDC1 protein.

Table 2. Phenotypes, genotypes, and demographic features of individuals with an *AHDC1* missense mutation

Patient ID	1	3	5	6	7	8	9	10
**Mutation**								
Nucleotide change	c.139C>T	c.1610G>A	c.1646G>A	c.1819G>A	c.2374G>C	c.4042T>C	c. 4370A>G	c.4432C>T
Protein change	p.Pro47Ser	p.Gly537Asp	p.Arg549His	p.Asp607Asn	p.Gly792Arg	p.Ser1348Pro	p.Asp1457Gly	p.Pro1478Ser
Age	14 years	10 years	6 years	23 years	12 years	10 years	2 years	11 years
Sex	M	F	F	M	F	M	F	F
Ethnicity	white	African American/white	white	white	white	white	NA	Latino/Hispanic
**Growth**								
Stature (percentile)	<10th	99th	>90th	43rd	99th	30th	1st	1st
Scoliosis	Y	N	N	N	N	N	NA	N
**Comprehensive skills and language**								
M-CHAT score	4	NA	NA	15	4		NA	0
Autism diagnosis	Y	N	N	Y	N	Y	NA	Y
Current language[a]	3	3	2	3	3	0	1	1
Age at first word	11 months	3 years	~2 years	2.5 years	2 years	NA	NA	2–3 years
Age using two words together	~2 years		~4 years	~12–13 years	not recalled		NA	NA
Age at following command	2 years		NA	NA	has trouble following command	not reported	NA	1.5 years
**Mobility**								
Hypotonia diagnosis	Y	N	N	Y	Y	Y	Y	Y
Independent walking	Y	Y	Y	Y	walking with support	Y		Y
Age at independent walking	~2 years	11 months	15 months	1.5 years		2 years		1 year

(*Continued on next page*)

Table 2. *Continued*

Patient ID	1	3	5	6	7	8	9	10
**Sleep/airway**								
Sleep apnea	N	Y	N	N	N			N
Using breathing support	N	Y (CPAP at night)	N	N	N			N
**Neurology**								
MRI	normal	NA	not done	abnormal	abnormal	abnormal	abnormal	abnormal
EEG	normal	NA	NA	NA	normal	NA	abnormal	normal
Seizure	Y	Y	NA	Y	Y	Y	Y	N
Age at first seizure	3 years	NA		22 years	2–3 years	6 years	3 days	NA
Ataxia	N	N	N	Y	Y			Y
**Vision**								
Wearing glasses or contacts	N	Y	N	N	Y	Y	N	N
Visual acuity	20/30	hyperopia, night blindness	normal	NA	NA	hypermetropia	hypermetropia	NA
Strabismus	N	N	N	N	N	Y	Y	N
**Dysmorphic features**								
Features	coarse facial features	long palpebral fissures, deep-set eyes, hypertelorism, macrocephaly, cleft palate	broad forehead, thin upper lip	macrocephaly (likely familial)	upslanted palpebral fissures, microcephaly, low-set ears	broad forehead, wide nasal bridge, brachycephaly, microtia, clinodactyly 5th finger, mild microcephaly	almond-shaped eyes, thin upper lip, brachycephaly, microcephaly, protuberant ears	upslanting palpebral fissures, microcephaly

Of the total of 10 individuals, five joined the XGS Registry and provided all available clinical data (individuals 1, 3, 6, 7, and 10). Partial data were available for three of the additional five known individuals (5, 8, 9). M-CHAT, Modified Checklist for Autism in Toddlers; CPAP, continuous positive airway pressure; MRI, magnetic resonance imaging; EEG, electroencephalogram; M, male; F, female; Y, yes; N, no; NA, not applicable.
[a]Current language: 0, no words; 1, <50 words; 2, no sentence but >50 words; and 3, full sentence >200 words.

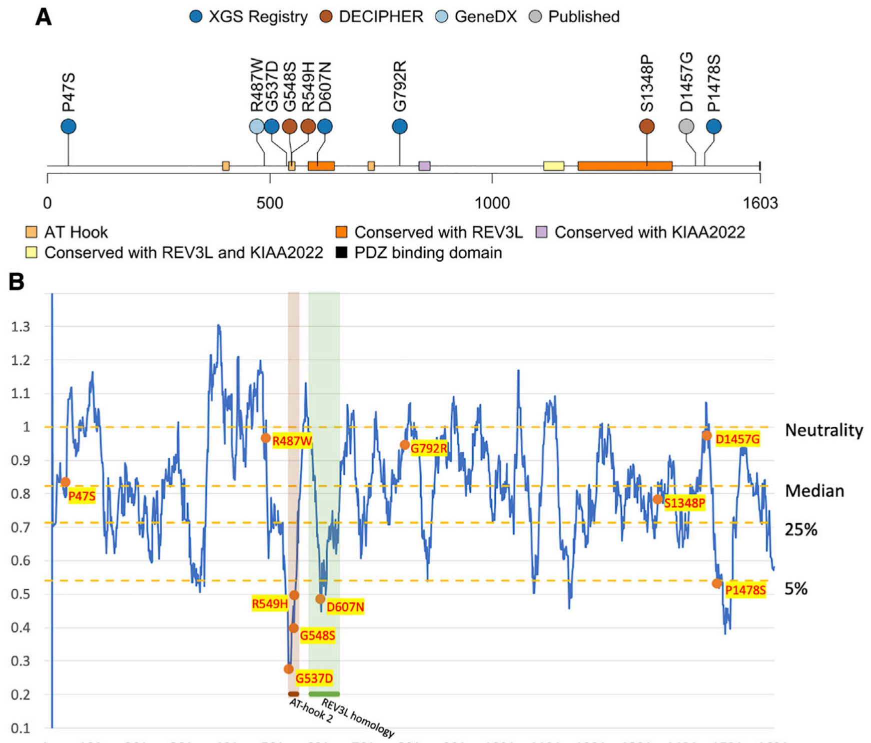

Figure 1. Recorded AHDC1 missense cases and protein sequence mutability
(A) A total of 10 individuals with *de novo* or suspected de novo missense mutations in *AHDC1* are shown.
(B) The *AHDC1* missense mutations are scored using the missense tolerance ratio score. A lower score indicates a higher intolerance to missense mutations based on sequence conservation of population controls from gnomAD.

Of note, two apparent clusters were observed, which included seven of the 10 missense variants. Cluster 1 contained four variants, spanning just 71 amino acid positions (537–607) within or flanking the region of the highly conserved AT-hook domain 2 and cluster 2, a conserved REV3L domain (Domain of Unknown Function 4683 [DUF4683]) (individuals 3–6). Cluster 2 consisted of three variants that spanned 131 residues near the C terminus of the protein, within or near a second domain that is conserved with REV3L (individuals 8–10) (Figure 1). One of these three variants (individual 9) is the mutation in the previously published report of the affected individual of Gumus.[6] Individual 10 bore a variant in close proximity, for which *de novo* status could not be inferred to provide supportive evidence due to the absence of paternal DNA. The two cluster regions are predicted to be intolerant to missense variation due to purifying selection (Figure 1B; Figure S1).

Three of the 10 missense variants fell outside the clusters. A variant at amino acid position 487 was within 51 residues of the first cluster, but where it "sits" in three-dimensional protein space and secondary and tertiary protein structure is unknown. The variants within individuals 1 (p.Pro47Ser) and 7 (p.Gly792Arg) did not cluster with other variants and the map to undefined AHDC1 protein regions, with no homology to other proteins.

Computational prediction of pathogenicity

Nine of 10 *de novo* or suspected *de novo* missense mutations in *AHDC1* considered here were predicted as LP using *in silico* pathogenicity scores including CADD and FATHMM-XF (Figure S1), with only the variant in individual 1 showing lower effect. However, variants within the two clusters described above tended to be within the top 10% of the highest pathogenicity score group. In contrast, the variants in the three individuals who were located outside the clusters consistently ranked lower in the calculated overall pathogenicity scores. Scoring using the REVEL meta predictor elevated the missense variants in the first cluster to the top 10%, compared to gnomAD controls.

Other mutation data

Other data, including variants in genes other than *AHDC1*, were considered as potential contributors to the clinical profiles of the individuals with missense *AHDC1* mutations who were in the XGS Registry. Individual 1, with a *de novo AHDC1* p.Pro47Ser variant, also harbored LP *de novo* missense variant c.10151A>G (p.Asp3384Gly) in FAT Atypical Cadherin 3 (*FAT3*). Although *FAT3* has not been definitively associated with a Mendelian disorder, recently a *FAT3* variant has been implicated as a potential contributor to autism spectrum disorder (ASD).[29] Further, the *AHDC1 de novo* missense mutation in individual 1 was classified as LB according to ACMG criteria. This classification was supported by the observation of different alleles at the same amino acid position in two individuals in the gnomAD database—although those alleles were not observed in the gnomAD reported "normal" (control) cohort and may also have had disease association.

Individual 2, with missense variant c.1459C>T (p.Arg487Trp), was potentially highly informative for this study, as the mutation occurred at position 487, which was near the proximal boundary of the cluster of four variants spanning amino acid positions 537–607. Consultation with the individual's caregivers revealed, however, that the initial genetic evaluation of the *AHDC1* missense mutation noted mosaicism, although further details were unavailable. Further, features that were atypical for XGS were noted (Supplemental notes). Overall, it was

Table 3. Additional genetic findings in individuals with *AHDC1 de novo* missense mutations

Case #	Gene(s)	Nucleotide change	Amino acid change	Zygosity	Inheritance pattern	gnomAD AC/AF	Predicted pathogenicity
1	*AHDC1*	c.139C>T	p.Pro47Ser	heterozygous	*de novo*	0/0	likely benign
	FAT3	c.10151A>G	p.Asp3384Gly	heterozygous	*de novo*	0/0	likely pathogenic
	SERPINA1	c.863A>G	p.Glu288Val	heterozygous	maternal	0/0	uncertain significance
3	*AHDC1*	c.1610G>A	p.Gly537Asp	heterozygous	*de novo*	0/0	likely pathogenic
	ANK3	c.6715C>T	p.Arg2239Cys	heterozygous	paternal	13/0.00005	uncertain significance
	APC2	c.4958G>A	p.Arg1653Gln	heterozygous	paternal	58/0.0004	uncertain significance
	C5orf42	c.8397A>C	p.Lys2799Asn	heterozygous	maternal	0/0	uncertain significance
	SON	c.313A>G	p.Thr105Ala	heterozygous	paternal	9/0.0004	uncertain significance
	TTC19	c.380A>G	p.Tyr127Cys	heterozygous	paternal	0/0	uncertain significance
	TPM3, C1orf189, C1orf43, UBAP2L, HAX1, MIR190B	microdeletion within 1q21.3	(154,150,447-154, 255,258)x1	heterozygous	unknown	NA	uncertain significance
6	*AHDC1*	c.1819G>A	p.Asp607Asn	heterozygous	*de novo*	0 / 0	likely pathogenic
	DNAH14	c.409C>T	p.Arg137*	heterozygous	paternal	198/0.0007	uncertain significance
	DNAH14	c.13548A>T	p.*4516Tyrfs*5	heterozygous	maternal	1,216/0.007	uncertain significance
7	*AHDC1*	c.2374G>C	p.Gly792Arg	heterozygous	*de novo*	0/0	uncertain significance
	NPHP1	microdeletion within 2q13	(110,199,004-110, 337,690)x1	heterozygous	paternal	NA	uncertain significance
	ATP11, CXorf661, MIR505	duplication within Xq27.1	(138,699,164-139, 089,567)x3	heterozygous	maternal	NA	uncertain significance
8[a]	*AHDC1*	c.4042T>C	p.Ser1348Pro	heterozygous	*de novo*	0/0	likely pathogenic
	HUWE1	c.9070G>A	p.Ala3024Thr	hemizygous	*de novo*	0/0	likely pathogenic
	NEB	c.9139C>A	p.His3047Asn	heterozygous	paternal	191/0.0005	benign
	NEB	c.7343G>A	p.Arg2448His	heterozygous	maternal	33/0.00008	benign

Data for individuals 1, 3, 6, and 7 were from the XGS Registry. Individual 10, also in the XGS Registry, did not report additional considered variants. AC/AF, allele count/allele frequency.
[a]Data for individual 8, not in the Registry, were provided with consent by the individual's health provider. Predicted pathogenicity was assessed using VarSome as described in Subjects and methods.

determined that the p.Arg487Trp change in this individual is not likely to be contributing to the phenotype, although it is possible that in other, non-mosaic individuals the variant may contribute to disease. If the mutation were not pathogenic, then the remaining four changes in the cluster region would span just 71 amino acids, including the critical AT-hook motif.

The diagnosis of individual 8, who harbored a COOH-terminal missense variant allele in *AHDC1* (p.Ser1348Pro) was confounded by the presence of a hemizygous LP *de novo* missense mutation in the HECT, UBA, and WWE Domain Containing E3 Ubiquitin Protein Ligase 1 (*HUWE1* c.9070G>A [p.Ala3024Thr]) gene. Missense mutations in *HUWE1* are highly constrained (missense *Z* score = 8.87); both SNV and duplication Copy Number Variation involving *HUWE1* are known causes of X-linked intellectual disability (ID; MIM: 309590).

No other potentially pathogenic variants were identified in other individuals in this study, although a series of variants were reviewed and determined to be VUS (Table 3).

Phenotypic spectrum of individuals with an *AHDC1* missense mutation

We previously defined five core and 12 secondary clinically observed phenotypic XGS features, based on the clinical presentations of 34 individuals with XGS due to truncating mutations in *AHDC1*.[1] The five core phenotypes were observed in >80% of XGS individuals and thus perhaps represent a clinical synopsis of the AD trait associated with this locus. These clinically observed findings include delayed motor milestones, ID, hypotonia, low muscle tone, and speech delay, potentially refining the core *AHDC1*-associated NDD trait. For the current study, we combined hypotonia and low muscle tone into a single category, although the two features were reported separately during most assessments. We compared the occurrence of these core and secondary phenotypes derived from individuals with truncating mutations to those with *AHDC1* missense mutations. These comparisons provided evidence that the spectrum of clinical manifestations of individuals reported with putatively pathogenic missense

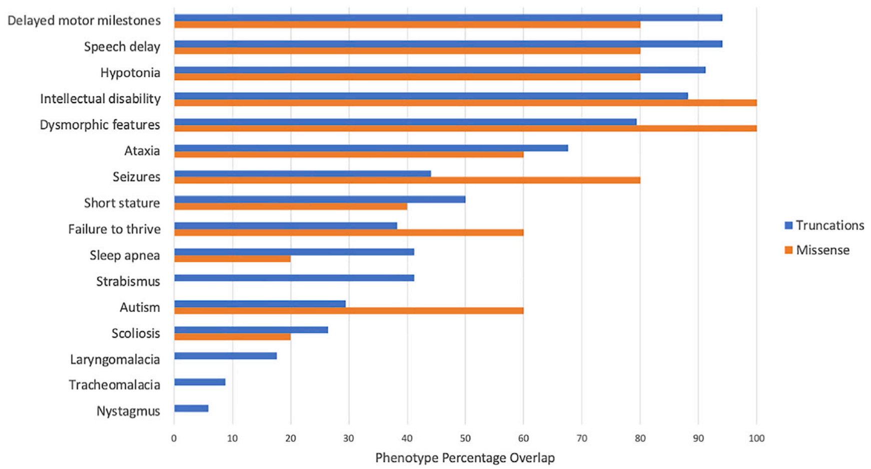

Figure 2. Comparison of XGS phenotypes
Data from 34 individuals with XGS due to truncating *AHDC1* mutations were compared with those from 5 individuals with suspected or confirmed *de novo* missense mutations in *AHDC1*, who have joined the XGS Registry.

mutations in *AHDC1* largely overlapped with those harboring truncating mutations (Figure 2).

Of note, all five individuals from the XGS Registry with missense variants had ID, and four of the five exhibited delayed motor milestones, speech delay, and a diagnosis of hypotonia/low muscle tone. Individuals in this study who were ascertained through public databases or previous studies did not have the same consistent phenotypic assessment information available as the information content available for those from the XGS Registry. Phenotypes for subject 2 were not available, and the DECIPHER individuals had limited phenotypic information upon query (Supplemental notes). However, the majority of cases had indicated phenotypes of ID and speech delay with the occurrence of other core phenotypes. An exception to the phenotypic spectrum associated with the missense mutations, compared to that of the truncating mutations, was that four of the five individuals in the XGS Registry with missense mutations reported seizures, compared to an incidence of approximately 50% in the truncation cases. As this feature is of high clinical relevance, we also examined the partial phenotype data available from three of the five external individuals not included in the XGS Registry. Overall, six of eight XGS individuals reported seizures, and data for one were not available (Table 2). Notably, data from individual 10, who had joined the XGS Registry and indicated "no" for this feature, initially indicated that the parents had suspected mild seizures. Subsequent physician records, however, indicated a series of normal EEGs and a record of no seizures. Overall, these data suggest a higher incidence of seizures in this group of individuals with missense *AHDC1* mutations, relative to truncation cases. It is not clear if this might represent a gain-of-function (GoF) versus loss-of-function (LoF) mutational effect.

Phenotypic clustering of individuals with missense mutations

To further investigate phenotypic similarities between individuals with a *de novo* missense versus a truncation mutation in *AHDC1*, a comparative analysis utilizing the 17 phenotypic terms collected from the XGS Registry was implemented. The 17 observed clinical phenotype features were converted into HPO terms and used to compare the phenotypic similarities among all the individuals with XGS, as well as the clinical manifestations of 3,464 human disease traits as defined by the clinical synopsis of individual entries from OMIM. Data from individuals with missense variants in *AHDC1* clustered together with those from individuals harboring truncating mutations (Figure 3A). In addition, individuals with a truncation or missense mutation were more similar to each other than to other diseases with similar phenotypes (Figure 3B). Collectively, by quantitative phenotyping and objective similarity searches, individuals with missense variants were phenotypically more similar to XGS due to truncating mutations, than to other disorders.

Discussion

The first 20 individuals reported with an XGS diagnosis bore *de novo* protein-truncating mutations, presumably LoF alleles, leading to speculation that *AHDC1* missense variants may not cause disease.[8] Alternative interpretations could be that missense variant alleles might potentially cause GoF versus LoF effects, or perhaps a different distinct rare disease trait. Truncating *AHDC1* mutations are also predominant among the more than 270 families with XGS now known worldwide, including 84 who have joined the XGS Registry. Nevertheless, there are at least 10 individuals who have been assigned an XGS diagnosis on the basis that they have *de novo* or suspected *de novo* missense mutations in *AHDC1*. Five of these 10 individuals with missense mutations have joined the XGS Registry and provided detailed clinical phenotyping records for further study and analyses. One additional family provided limited clinical data, and information from the four remaining individuals was drawn from DECIPHER or publication. No biomarker or other laboratory assays are yet available to directly evaluate the biological effect of the amino acid substitutions on AHDC1 protein function, and therefore these molecular diagnoses rely considerably on the *de novo* status of the changes and the absence of other variants/loci that parsimoniously explain the observed clinical features. Further consideration of the genetic and phenotypic data for these reported cases affords

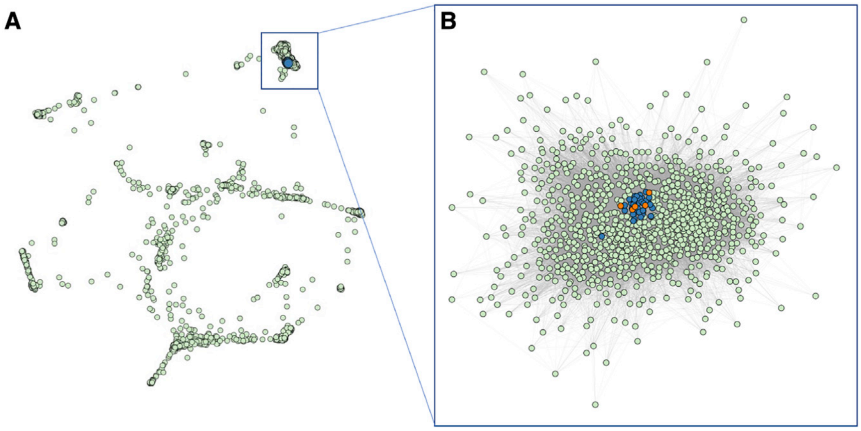

Figure 3. Phenotypic similarity network between individuals with *AHDC1* variants and OMIM diseases

(A) Clustering of individuals with an *AHDC1* missense mutation or truncation mutation with 3,464 diseases reported to OMIM based on phenotype similarity illustrated by orange dots, blue dots, and green dots, respectively.

(B) Reclustered OMIM disease nodes with at least one connection and similarity >0.1 to individuals with an *AHDC1* missense or truncation mutation.

the opportunity to substantiate that missense mutations can cause XGS. As also suggested by our XGS data analysis and the *FAT3* variation (individual 1), correlation with clinical phenotyping and expected observations may be useful in molecular diagnostic interpretation, and the presence of multilocus pathogenic variation needs to be considered in the molecular differential.[30]

The amino acid positions of the 10 missense *AHDC1* variants reported as underlying XGS suggest a clustering of events in two primary sequence regions of the protein. One cluster was striking, as it contained 4/10 mutations within 71 amino acid residues—less than 5% of the protein. This cluster also contained an AT-hook binding domain, with two of the missense mutations in the core AT-hook 12-amino-acid motif.[12] The second, more broadly defined cluster contained three missense variants and spanned 131 amino acid residues—about 8% of the protein. The three remaining mutations did not map to any defined cluster or associate with regions of strong conservation or computationally defined domains. Although the structure of AHDC1 is not solved, and 3D protein effects cannot yet be considered, these data suggest that the variants that arise in either of the two clusters are potentially more likely to perturb normal protein function.

Algorithmic prediction of the likelihood of the missense mutations being damaging to the AHDC1 protein also distinguished variants within the two clusters from the mutations that occurred in surrounding regions. While all 10 of the missense *AHDC1* variants in this study passed the likelihood threshold for deleteriousness using the CADD scores (21.5 to 28.6) to predict their effects, those outside of the clusters had generally lower scores (21.5 to 24.8). FATHMM-XF analysis further differentiated the two classes of variants, with all mutations in the clusters reported as LP and the three additional variants with the lowest scores for pathogenicity (Figure S1C). We also observed the general trend of most of the clustered mutations being in regions of very low MTR scores, indicating high intolerance to change (Figure 1B). Overall, the data and the computational *in silico* analyses support the general model of at least two mutation-sensitive regions within the AHDC1 protein.

The information content of quantitative phenotyping and comparison of phenotypes from individuals with *AHDC1* mutations did not initially provide additional insight into the true impact of the missense mutations. The same overall pattern was observed in individuals with missense mutations versus those with truncating mutations, and when HPO terms were analyzed both groups had essentially the same distance from other neurological conditions in OMIM. This was an expected result, as one of the criteria for the assignment of an XGS diagnosis was having a similar phenotype to the already-reported individuals. The important question of whether there are individuals who harbor missense *AHDC1* alleles with lower functional impact than any of the missense mutations reported here is unanswered. Such individuals may have much milder phenotypes and may not present for clinical evaluation. Hypomorphic alleles may also lead to disease traits with incomplete penetrance, leading to under-ascertainment of individuals with inherited variant alleles. Nevertheless, the quantitative analyses of the XGS Registry data did reveal the association of reported seizures with these missense cases, and that finding was distinguishable from what has been reported for truncating alleles. We speculate that these findings might implicate some potential GoF effects of *AHDC1* missense variants.

Together, the data here suggest guidelines for consideration of the possible pathogenicity of newly observed missense variants in *AHDC1* in individuals with XGS-like phenotypes and in the absence of other genomic variants that explain an individual's clinical presentation. Foremost, established guidelines from the ACMG should be used to guide variant interpretation. These consider most factors that are contained in the current discussion, including inheritance pattern and computational predictions of likelihood of damaging effects. The *de novo* status of any *AHDC1* missense variant should be considered as a highly important factor for diagnosis, as there is as yet no evidence for any transmitted variant-causing disease. Moreover, the position of the mutation might be considered, with variant alleles mapping within either of the two cluster regions prioritized for assignment as disease

causing. There are many caveats to these guidelines, including the imprecision of the knowledge of the cluster boundaries and the likelihood that with improved understanding of AHDC1 structure and function some missense changes outside the clusters may be determined to be pathogenic. Nevertheless, it is clear from both the population data that include many variants in reportedly “normal” individuals and the different presentation of the variant data from individuals 1, 2, and 7 in this study that pathogenicity cannot be confidently asserted from *de novo* status alone. Further, the study underscores the high priority for both accrual of larger datasets and development of independent functional assays for the protein. One further potential insight from our missense clinical data regarding missense constraint and gnomAD-facilitated variant interpretation, and as has also been gleaned from studies of premature truncating codon (PTC) interpretation, there may be limitations to gnomAD-assisted variant interpretation when potentially dealing with GoF mutation effects.[31–33]

The mechanism by which *AHDC1* mutations result in XGS is unknown, and there have been prior suggestions of both haploinsufficiency and dominant negative effects.[1,34] Missense mutations are expected to result in full-length protein products and are therefore more likely to act in dominant negative (antimorphic) or other GoF (neomorphic and hypermorphic) manners. Consequently, the report here and from prior missense mutations supports the dominant negative model, but it does not exclude that both haploinsufficiency (LoF) and dominant negative (GoF) mechanisms could be at play, depending on the primary mutation.

There are a growing number of early-onset severe neurological Mendelian diseases for which, like XGS, a known gene with *de novo* truncating mutations leads to disease.[35–37] There is, however, a paucity of well-studied examples where such loci are also damaged by missense variants and yield related phenotypes. This is in part likely due to the complexity of determining causation for missense variants in disease diagnosis. Where previous work has identified pathogenic missense variants in genes that are also associated with autosomal dominant disorders (e.g., *NOTCH3*), the enrichment of missense variants in conserved protein domains has been noted.[38] This trend is consistent with observations here of missense mutations clustering within and near the conserved AT-hook 2 domain and the REV3L homology domain (DUF4683) in *AHDC1*.

In summary, we report quantitative phenotyping and analyses in 10 *AHDC1* missense mutations—five novel putative XGS molecular diagnoses and five reported mutations. These novel missense *AHDC1* variants newly described in this study provide confirmatory supportive evidence that some missense variants in *AHDC1* can cause XGS. Quantitative clinical phenotyping reveals missense alleles share a core monoallelic NDD autosomal dominant trait phenotype with that observed in association with LoF variant alleles but suggests that seizures may more likely be observed in association with missense alleles. To what extent such findings might implicate potential GoF effects remains to be determined by more extensive studies of *AHDC1* allelic series.

Data and code availability

The published article includes all datasets generated or analyzed during the study. Additional data that support the findings of this study are available upon request from the corresponding author, subject to privacy or ethical restrictions. The variants reported here are deposited in the ClinVar database.

Supplemental information

Supplemental information can be found online at https://doi.org/10.1016/j.xhgg.2021.100049.

Acknowledgments

This work would have not been possible without the contributions of family members participating in the AHDC1 Xia-Gibbs Registry. We also thank Drs. Gail E. Herman and Jane Hurst for case referral. The scientific and experimental work contained herein were funded in part by the NHGRI awards to R.A.G. (HG008898) and J.R.L. (NHGRI/NHLBI UM1 HG006542; NINDS R35 NS105078) and by private donations. V.C. was supported by the training fellowship from the NLM Training Program in Biomedical Informatics & Data Science (T15LM007093). H.L. was partially supported by an award from the Xia-Gibbs Society. J.E.P. was supported by NHGRI K08 HG008986. J.W.I. was supported by the Morton S. and Henrietta K. Sellner Professorship. This study makes use of data generated by the DECIPHER community. A full list of centers who contributed to the generation of the data is available from DECIPHER and via email from contact@deciphergenomics.org. Funding for the DECIPHER project was provided by Wellcome. The Deciphering Developmental Disorders (DDD) study presents independent research commissioned by the Health Innovation Challenge Fund (HICF-1009-003), a parallel funding partnership between Wellcome and the Department of Health, and the Wellcome Sanger Institute (WT098051). The views expressed in this publication are those of the author(s) and not necessarily those of Wellcome or the Department of Health. The study has UK Research Ethics Committee approval (10/H0305/83, granted by the Cambridge South REC, and GEN/284/12 granted by the Republic of Ireland REC). The research team acknowledges the support of the National Institute for Health Research through the Comprehensive Clinical Research Network.

Declaration of interests

J.R.L. has stock ownership in 23andMe, is a paid consultant for the Regeneron Genetics Center, and is a coinventor on multiple US and European patents related to molecular diagnostics for inherited neuropathies, eye diseases, and bacterial genomic fingerprinting. The Department of Molecular and Human Genetics at Baylor College of Medicine derives revenue from the chromosomal microarray analysis (CMA) and clinical exome sequencing (cES) offered in the Baylor Genetics (BG) Laboratory. J.R.L. serves on the Scientific Advisory Board of BG. J.F.’s spouse is Founder and Principal of Friedman Bioventure, which holds a variety of

publicly traded and private biotechnology interests. K.M. is an employee of GeneDx, Inc. All other authors declare no competing interests.

Received: May 21, 2021
Accepted: August 4, 2021

Web resources

ClinVar, https://www.ncbi.nlm.nih.gov/clinvar/
DECIPHER, https://www.deciphergenomics.org/about/stats
OMIM, https://omim.org/

References

1. Khayat, M.M., Li, H., Chander, V., Hu, J., Hansen, A.W., Li, S., Traynelis, J., Shen, H., Weissenberger, G., Stossi, F., et al. (2021). Phenotypic and protein localization heterogeneity associated with AHDC1 pathogenic protein-truncating alleles in Xia–Gibbs syndrome. Hum. Mutat *42*, 577–591.
2. Cardoso-Dos-Santos, A.C., Oliveira Silva, T., Silveira Faccini, A., Woycinck Kowalski, T., Bertoli-Avella, A., Morales Saute, J.A., Schuler-Faccini, L., and de Oliveira Poswar, F. (2020). Novel *AHDC1* Gene Mutation in a Brazilian Individual: Implications of Xia-Gibbs Syndrome. Mol. Syndromol. *11*, 24–29.
3. Cheng, X., Tang, F., Hu, X., Li, H., Li, M., Fu, Y., Yan, L., Li, Z., Gou, P., Su, N., et al. (2019). Two Chinese Xia-Gibbs syndrome patients with partial growth hormone deficiency. Mol. Genet. Genomic Med. *7*, e00596.
4. Díaz-Ordoñez, L., Ramirez-Montaño, D., Candelo, E., Cruz, S., and Pachajoa, H. (2019). Syndromic Intellectual Disability Caused by a Novel Truncating Variant in AHDC1: A Case Report. Iran. J. Med. Sci. *44*, 257–261.
5. García-Acero, M., and Acosta, J. (2017). Whole-Exome Sequencing Identifies a de novo *AHDC1* Mutation in a Colombian Patient with Xia-Gibbs Syndrome. Mol. Syndromol. *8*, 308–312.
6. Gumus, E. (2020). Extending the phenotype of Xia-Gibbs syndrome in a two-year-old patient with craniosynostosis with a novel de novo AHDC1 missense mutation. Eur. J. Med. Genet. *63*, 103637.
7. He, P., Yang, Y., Zhen, L., and Li, D.-Z. (2020). Recurrent hypoplasia of corpus callosum as a prenatal phenotype of Xia-Gibbs syndrome caused by maternal germline mosaicism of an AHDC1 variant. Eur. J. Obstet. Gynecol. Reprod. Biol. *244*, 208–210.
8. Jiang, Y., Wangler, M.F., McGuire, A.L., Lupski, J.R., Posey, J.E., Khayat, M.M., Murdock, D.R., Sanchez-Pulido, L., Ponting, C.P., Xia, F., et al. (2018). The phenotypic spectrum of Xia-Gibbs syndrome. Am. J. Med. Genet. A. *176*, 1315–1326.
9. Mubungu, G., Makay, P., Boujemla, B., Yanda, S., Posey, J.E., Lupski, J.R., Bours, V., Lukusa, P., Devriendt, K., and Lumaka, A. (2021). Clinical presentation and evolution of Xia-Gibbs syndrome due to p.Gly375ArgfsTer3 variant in a patient from DR Congo (Central Africa). Am. J. Med. Genet. A. *185*, 990–994.
10. Murdock, D.R., Jiang, Y., Wangler, M., Khayat, M.M., Sabo, A., Juusola, J., McWalter, K., Schatz, K.S., Gunay-Aygun, M., and Gibbs, R.A. (2019). Xia-Gibbs syndrome in adulthood: a case report with insight into the natural history of the condition. Cold Spring Harb. Mol. Case Stud. *5*, a003608.
11. Ritter, A.L., McDougall, C., Skraban, C., Medne, L., Bedoukian, E.C., Asher, S.B., Balciuniene, J., Campbell, C.D., Baker, S.W., Denenberg, E.H., et al. (2018). Variable Clinical Manifestations of Xia-Gibbs syndrome: Findings of Consecutively Identified Cases at a Single Children's Hospital. Am. J. Med. Genet. A. *176*, 1890–1896.
12. Xia, F., Bainbridge, M.N., Tan, T.Y., Wangler, M.F., Scheuerle, A.E., Zackai, E.H., Harr, M.H., Sutton, V.R., Nalam, R.L., Zhu, W., et al. (2014). De novo truncating mutations in AHDC1 in individuals with syndromic expressive language delay, hypotonia, and sleep apnea. Am. J. Hum. Genet. *94*, 784–789.
13. Yang, S., Li, K., Zhu, M.-M., Yuan, X.-D., Jiao, X.-L., Yang, Y.-Y., Li, J., Li, L., Zhang, H.N., Du, Y.H., et al. (2019). Rare Mutations in AHDC1 in Patients with Obstructive Sleep Apnea. BioMed Res. Int. *2019*, 5907361.
14. Yang, H., Douglas, G., Monaghan, K.G., Retterer, K., Cho, M.T., Escobar, L.F., Tucker, M.E., Stoler, J., Rodan, L.H., Stein, D., et al. (2015). De novo truncating variants in the AHDC1 gene encoding the AT-hook DNA-binding motif-containing protein 1 are associated with intellectual disability and developmental delay. Cold Spring Harb. Mol. Case Stud. *1*, a000562.
15. Thul, P.J., Åkesson, L., Wiking, M., Mahdessian, D., Geladaki, A., Ait Blal, H., Alm, T., Asplund, A., Björk, L., Breckels, L.M., et al. (2017). A subcellular map of the human proteome. Science *356*, eaal3321.
16. Uhlén, M., Fagerberg, L., Hallström, B.M., Lindskog, C., Oksvold, P., Mardinoglu, A., Sivertsson, Å., Kampf, C., Sjöstedt, E., Asplund, A., et al. (2015). Proteomics. Tissue-based map of the human proteome. Science *347*, 1260419.
17. Karczewski, K.J., Francioli, L.C., Tiao, G., Cummings, B.B., Alföldi, J., Wang, Q., Collins, R.L., Laricchia, K.M., Ganna, A., Birnbaum, D.P., et al.; Genome Aggregation Database Consortium (2020). The mutational constraint spectrum quantified from variation in 141,456 humans. Nature *581*, 434–443.
18. Miller, K.A., Twigg, S.R.F., McGowan, S.J., Phipps, J.M., Fenwick, A.L., Johnson, D., Wall, S.A., Noons, P., Rees, K.E., Tidey, E.A., et al. (2017). Diagnostic value of exome and whole genome sequencing in craniosynostosis. J. Med. Genet. *54*, 260–268.
19. Karaca, E., Posey, J.E., Coban Akdemir, Z., Pehlivan, D., Harel, T., Jhangiani, S.N., Bayram, Y., Song, X., Bahrambeigi, V., Yuregir, O.O., et al. (2018). Phenotypic expansion illuminates multilocus pathogenic variation. Genet. Med. *20*, 1528–1537.
20. Firth, H.V., Richards, S.M., Bevan, A.P., Clayton, S., Corpas, M., Rajan, D., Van Vooren, S., Moreau, Y., Pettett, R.M., and Carter, N.P. (2009). DECIPHER: Database of Chromosomal Imbalance and Phenotype in Humans Using Ensembl Resources. Am. J. Hum. Genet *84*, 524–533.
21. Harris, P.A., Taylor, R., Minor, B.L., Elliott, V., Fernandez, M., O'Neal, L., McLeod, L., Delacqua, G., Delacqua, F., Kirby, J., Duda, S.N.; and The REDCap consortium (2019). Building an international community of software platform partners. J. Biomed. Inform *95*, 103208.
22. Sobreira, N., Schiettecatte, F., Valle, D., and Hamosh, A. (2015). GeneMatcher: a matching tool for connecting investigators with an interest in the same gene. Hum. Mutat. *36*, 928–930.
23. Köhler, S., Gargano, M., Matentzoglu, N., Carmody, L.C., Lewis-Smith, D., Vasilevsky, N.A., Danis, D., Balagura, G., Baynam, G., Brower, A.M., et al. (2021). The human phenotype ontology in 2021. Nucleic Acids Res. *49* (D1), D1207–D1217.

24. Traynelis, J., Silk, M., Wang, Q., Berkovic, S.F., Liu, L., Ascher, D.B., Balding, D.J., and Petrovski, S. (2017). Optimizing genomic medicine in epilepsy through a gene-customized approach to missense variant interpretation. Genome Res. *27*, 1715–1729.
25. Rentzsch, P., Witten, D., Cooper, G.M., Shendure, J., and Kircher, M. (2019). CADD: predicting the deleteriousness of variants throughout the human genome. Nucleic Acids Res. *47* (D1), D886–D894.
26. Rogers, M.F., Shihab, H.A., Mort, M., Cooper, D.N., Gaunt, T.R., and Campbell, C. (2018). FATHMM-XF: accurate prediction of pathogenic point mutations via extended features. Bioinformatics *34*, 511–513.
27. Ioannidis, N.M., Rothstein, J.H., Pejaver, V., Middha, S., McDonnell, S.K., Baheti, S., Musolf, A., Li, Q., Holzinger, E., Karyadi, D., et al. (2016). REVEL: An Ensemble Method for Predicting the Pathogenicity of Rare Missense Variants. Am. J. Hum. Genet. *99*, 877–885.
28. Kopanos, C., Tsiolkas, V., Kouris, A., Chapple, C.E., Albarca Aguilera, M., Meyer, R., and Massouras, A. (2019). VarSome: the human genomic variant search engine. Bioinformatics *35*, 1978–1980.
29. Baldwin, I., Shafer, R.L., Hossain, W.A., Gunewardena, S., Veatch, O.J., Mosconi, M.W., and Butler, M.G. (2021). Genomic, clinical, and behavioral characterization of 15q11.2 bp1-bp2 deletion (burnside-butler) syndrome in five families. Int. J. Mol. Sci. *22*, 1–24.
30. Posey, J.E., Harel, T., Liu, P., Rosenfeld, J.A., James, R.A., Coban Akdemir, Z.H., Walkiewicz, M., Bi, W., Xiao, R., Ding, Y., et al. (2017). Resolution of Disease Phenotypes Resulting from Multilocus Genomic Variation. N. Engl. J. Med. *376*, 21–31.
31. Poli, M.C., Ebstein, F., Nicholas, S.K., de Guzman, M.M., Forbes, L.R., Chinn, I.K., Mace, E.M., Vogel, T.P., Carisey, A.F., Benavides, F., et al.; Undiagnosed Diseases Network members (2018). Heterozygous Truncating Variants in POMP Escape Nonsense-Mediated Decay and Cause a Unique Immune Dysregulatory Syndrome. Am. J. Hum. Genet. *102*, 1126–1142.
32. Bayram, Y., White, J.J., Elcioglu, N., Cho, M.T., Zadeh, N., Gedikbasi, A., Palanduz, S., Ozturk, S., Cefle, K., Kasapcopur, O., et al.; Baylor-Hopkins Center for Mendelian Genomics (2017). REST Final-Exon-Truncating Mutations Cause Hereditary Gingival Fibromatosis. Am. J. Hum. Genet. *101*, 149–156.
33. Coban-Akdemir, Z., White, J.J., Song, X., Jhangiani, S.N., Fatih, J.M., Gambin, T., Bayram, Y., Chinn, I.K., Karaca, E., Punetha, J., et al.; Baylor-Hopkins Center for Mendelian Genomics (2018). Identifying Genes Whose Mutant Transcripts Cause Dominant Disease Traits by Potential Gain-of-Function Alleles. Am. J. Hum. Genet. *103*, 171–187.
34. Quintero-Rivera, F., Xi, Q.J., Keppler-Noreuil, K.M., Lee, J.H., Higgins, A.W., Anchan, R.M., Roberts, A.E., Seong, I.S., Fan, X., Lage, K., et al. (2015). MATR3 disruption in human and mouse associated with bicuspid aortic valve, aortic coarctation and patent ductus arteriosus. Hum. Mol. Genet. *24*, 2375–2389.
35. Yang, Y., Muzny, D.M., Reid, J.G., Bainbridge, M.N., Willis, A., Ward, P.A., Braxton, A., Beuten, J., Xia, F., Niu, Z., et al. (2013). Clinical whole-exome sequencing for the diagnosis of mendelian disorders. N. Engl. J. Med. *369*, 1502–1511.
36. Bamshad, M.J., Shendure, J.A., Valle, D., Hamosh, A., Lupski, J.R., Gibbs, R.A., Boerwinkle, E., Lifton, R.P., Gerstein, M., Gunel, M., et al.; Centers for Mendelian Genomics (2012). The Centers for Mendelian Genomics: a new large-scale initiative to identify the genes underlying rare Mendelian conditions. Am. J. Med. Genet. A. *158A*, 1523–1525.
37. Posey, J.E., O'Donnell-Luria, A.H., Chong, J.X., Harel, T., Jhangiani, S.N., Coban Akdemir, Z.H., Buysek, S., Pehlivan, D., Carvalho, C.M.B., Baxter, S., et al. (2019). Insights into genetics, human biology and disease gleaned from family based genomic studies. Genet. Med *21*, 798–812.
38. Turner, T.N., Douville, C., Kim, D., Stenson, P.D., Cooper, D.N., Chakravarti, A., and Karchin, R. (2015). Proteins linked to autosomal dominant and autosomal recessive disorders harbor characteristic rare missense mutation distribution patterns. Hum. Mol. Genet *24*, 5995–6002.

HGGA, Volume *2*

Supplemental information

AHDC1 missense mutations in Xia-Gibbs syndrome

Michael M. Khayat, Jianhong Hu, Yunyun Jiang, He Li, Varuna Chander, Moez Dawood, Adam W. Hansen, Shoudong Li, Jennifer Friedman, Laura Cross, Emilia K. Bijlsma, Claudia A.L. Ruivenkamp, Francis H. Sansbury, Jeffrey W. Innis, Jessica Omark O'Shea, Qingchang Meng, Jill A. Rosenfeld, Kirsty McWalter, Michael F. Wangler, James R. Lupski, Jennifer E. Posey, David Murdock, and Richard A. Gibbs

Supplemental Information:

Supplementary Figure 1: Evaluation of the frequency and pathogenicity of missense mutations compared to gnomAD controls. (A) Allele frequency vs protein codon for *AHDC1* missense mutations occurring in gnomAD controls. Analysis of individuals with *de novo* or suspected *de novo* missense mutations using pathogenicity scores from CADD (B), FATHMM-XF (C) and REVEL (D); gnomAD controls are displayed as grey dots and cases as red dots.

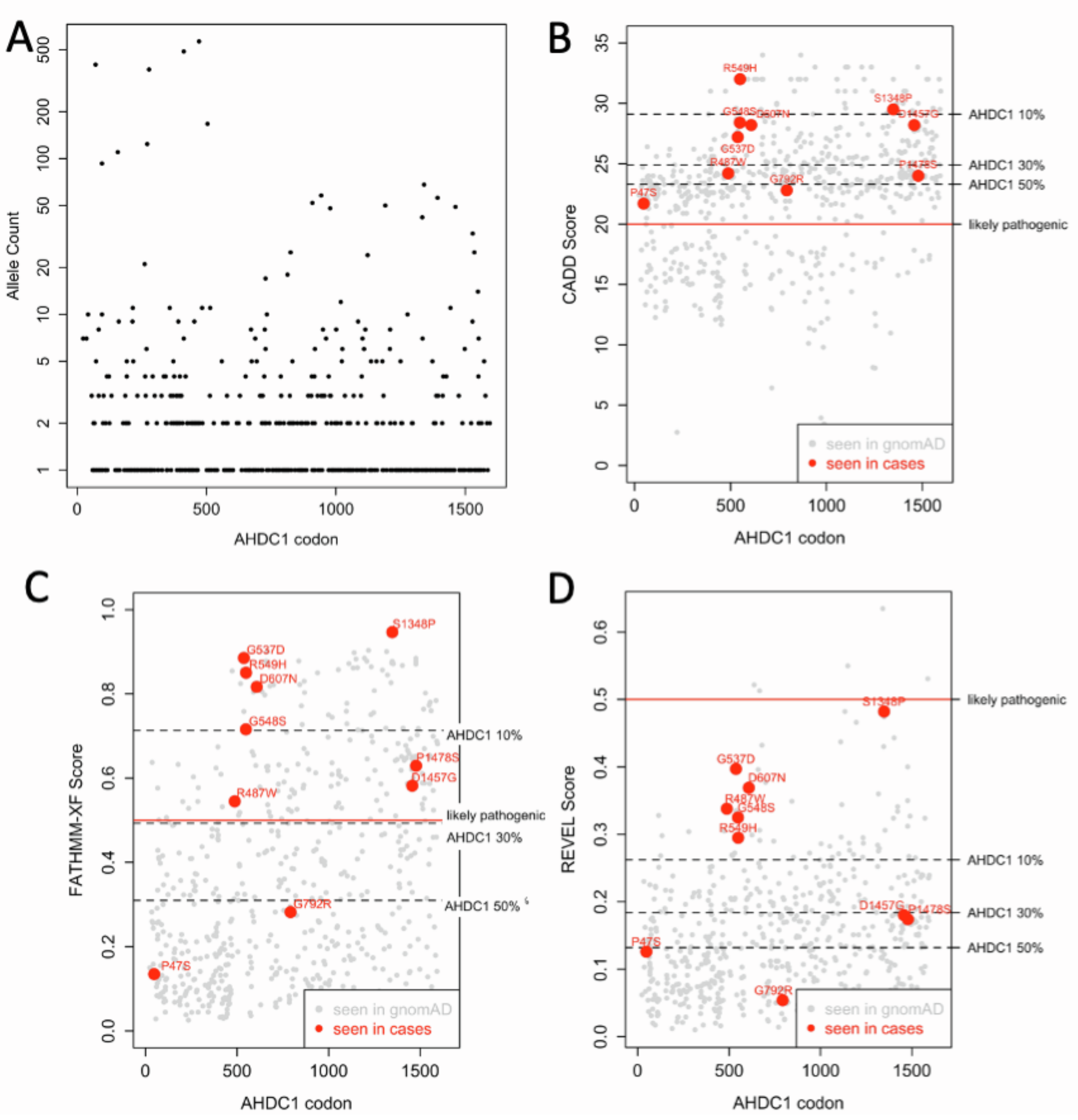

Supplemental Table 1. The missense variants in the *AHDC1* gene were classified using the ACMG standards and guidelines. PS2, PM2 and PP3 are the categories that can be applied to interpret the variants. PS2: *de novo* in a patient with the disease with both maternity and paternity confirmed and no family history, a strong evidence of pathogenicity. PM2: extremely low frequency in general population database gnomAD, a moderate evidence of pathogenicity. PP3: multiple lines of computational evidence support a deleterious effect on the gene or gene product, a supporting evidence of pathogenicity.

Case #	Nucleotide change	Protein change	ACMG classification
1	c.139C>T	p.Pro47Ser	Likely Benign
2	c.1459C>T	p.Arg487Trp	Likely Pathogenic
3	c.1610G>A	p.Gly537Asp	Likely Pathogenic
4	c.1642G>A	p.Gly548Ser	Likely Pathogenic
5	c.1646G>A	p.Arg549His	Likely Pathogenic
6	c.1819G>A	p.Asp607Asn	Likely Pathogenic
7	c.2374G>C	p.Gly792Arg	Uncertain Significance
8	c.4042T>C	p.Ser1348Pro	Likely Pathogenic
9	c.4370A>G	p.Asp1457Gly	Likely Pathogenic
10	c.4432C>T	p. Pro1478Ser	Uncertain Significance

Case Reports:

Case 1: is a 14-year-old male with cognitive developmental delay, speech delay, myopathy, coarse facial features, brachydactyly, small hands and feet, history of torticollis and precocious pubarche.

The patient has recurrent respiratory infections since 1 week old. Whole body bone scan by nuclear imaging at 12 years showed that he has skeletal dysplasia, including scoliosis, pectus carinatum, clubbed fingers, thickening of large bones, shortened bilateral femoral neck, enlarged and broad vertebrae, broad long bones, and short segment syrinx. X-ray and MRI showed an enchondroma in the left humerus. He was diagnosed with elevated liver enzymes at 11 years. He also has other chronic phenotypes including seasonal allergies, asthma, acid reflux, constipation and insomnia.

Neurology: The patient had a normal brain magnetic resonance imaging (MRI) at 3 years. He has sensory issues and was diagnosed with dysautonomia at 12 years.

Behavior: He has anxiety and obsessive-compulsive disorder. He has some autistic-like characteristics but was not diagnosed with autism. He has echolalia and will not answer questions without repeating the phrase that is stated to him.

Sequencing studies: Exome sequencing identified a *de novo* c.139C>T (p.Pro47Ser) variant in *AHDC1*. Two other variants in *FAT3* (c.10151A>G, p.Asp3384Gly; *de novo*) and *SERPINA1* (c.10151A>G, p. Glu288Val; maternally inherited) genes were also identified.

Referral: Direct contact by patient family/self-referral. This family consented to participation in the XGS Registry.

Case 2:

This individual has a neurodegenerative phenotype characterized by generalized dystonia and imaging showing basal ganglia mineralization. Consultation with the caregiver and the referring physician identified that while XGS was considered, the atypical clinical phenotype in conjunction with the mosaic nature of the variant ruled out the XGS diagnosis.

Sequencing studies: Exome sequencing identified a mosaic *de novo* c.1459C>T (p.Arg487Trp) variant in *AHDC1*.

Referral: Direct contact by individual's caregiver and referring physician. This family declined full participation in the XGS Registry, however agreed to share the data included in this publication.

Case 3: is a 10-year-old female with bifid uvula, learning delays, obesity, tall stature, and a questionable submucous cleft palate. The patient was born at 35 weeks of gestation via spontaneous vaginal delivery after an unremarkable pregnancy. She never passed newborn hearing screening. She smiled at 4 months of age, rolled over at 3 months of age and first walked at 11 months old. Her first words came at 3 years of age, which was likely helped by the placement of pressure equalization tubes.

The patient is 160.10 cm (> 99.9 %ile) and 114.20 kg (99.99 %ile) with body mass index (BMI) of 44.55 kg/m^2 at 10 years of age.

Neurology: This patient has not been evaluated by neurology, nor has she had a brain MRI.

Behavior: This patient has learning problems and a history of speech delays and food-seeking behavior. She does have behavior problems including aggression.

Sequencing studies:

This patient initially had a microarray, which identified a heterozygous ~105kb deletion within chromosome band 1q21.3 that contains exons 1 and 2 the *TPM3* gene, entire coding regions of *C1orf189*, *C1orf43*, *UBAP2L* and *HAX1* genes. The patient was also negative for Fragile X and Prader-Willi testing. A clinical feature-driven NGS panel testing identified a *de novo AHDC1* c.1610G>A (p.Gly537Asp) mutation in this patient. Other possible candidates are listed in Table 3.

Referral: This family consented to participation in the XGS Registry. Patient phenotype and case report were provided by caregiver.

Case 4: is an 11-year-old male registered in DECIPHER with learning and developmental delays. The patient was reported to have decreased fetal movement. Certain musculoskeletal abnormalities were reported including a positional foot deformity and congenital muscular torticollis.

Neurology: Cognitive impairment, delayed speech and language development and developmental regression.

Sequencing studies: A *de novo* missense variant was reported in *AHDC1* (c.1642G>A, p.Gly548Ser), initially identified by WGS and confirmed by targeted *AHDC1* sequencing.

Referral: Data described are from DECIPHER – consent was obtained directly from the parent for inclusion of this entry in this publication.

Case 5: is a 6-year-old girl, born as the first child of healthy non-consanguineous White parents. Her younger brother is healthy, and the family history is unremarkable. She was born full term after an uncomplicated pregnancy by vacuum-assisted vaginal delivery and weighed 4850 grams. Delivery was complicated by shoulder dystocia, resulting in obstetric brachial plexus injury (Erb's palsy). In the first year she was unable to use her right arm properly, but after extensive physiotherapy her palsy fully recovered.

The patient's motor development was within normal limits. She walked at 15 months. She was in good health, and she had normal hearing and vision. She was referred to the audiologic department at the age of 2y8m for evaluation of speech delay. She spoke her first word around 14 months but hardly had any speech at age 3. Her overall development was delayed. A nonverbal IQ test (SON-R 2-8) at 3 years of age showed that she functioned at a level of 1.5 years. She could talk in short

sentences at the age of 5. She could count and had learned to write numbers. She had just started to learn to read, and she could recognize the alphabet.

At 3 years and 9 months of age her height was 105.5 cm (+0.6 SDS), weight 17.4 kg (+0.3 SDS for height) with head circumference of 51 cm (+0.7 SDS). She has a wide forehead with a metopic ridge and a thin upper lip but is otherwise not dysmorphic. Apart from tapering fingers, she had no abnormalities of hands and feet.

Neurology: The patient did not have a brain MRI.

Behavior: The patient has no behavioral problems.

Sequencing studies: This patient was first seen by a clinical geneticist at age 3 years 9 months. SNP array (CytoScan HD Array Affymetrix) showed 2 copy number variants that were interpreted as LB variants. She has a small interstitial deletion of chromosome 4q of at least 261.2 kb (200 probes) from 98,349,035 bp to 98,610,262 bp (Hg19/GRCh37). The deletion includes one coding gene (*STPG2*), which is not linked to human disease. Furthermore, she has a small interstitial duplication of chromosome 12q of at least 358.5 kb (240 probes), from 123,656,808 bp to 124,015,352 bp. The duplication includes 8 coding genes (*MPHOSPH9*, *CDK2AP1*, *C12orf65*, *SBNO1*, *RILPL2*, *SETD8*, *SNRNP35*, and *RILPL1*). Karyotype: 46, XY; arr[GRCh37] 4q22.3(98349035_98610262)x1, 12q24.31(123656808_124015352)x3. Exome sequencing identified a *de novo AHDC1* c.1646G>A (p.Arg549His) mutation.

Referral: This case was included in DECIPHER database. Patient caregiver provided patient phenotype and case report.

Case 6: is a 23-year-old male with autism spectrum disorder, speech delay and intellectual disability. The major clinical features of this individual are listed in Table 2. He was born at full term with unremarkable pregnancy history. He was referred to a child development clinic at the age of 15 months for not walking or standing independently. He started walking without support at 18 months. He had his first word at approximately 2.5 years of age and did not start using two-word sentences until 12-13yo. He has severe impairment of receptive language.

Patient 6 is of average stature. He has a prominent sacral dimple.

Neurology: The patient had multiple MRIs performed. The first one was performed at the age of 14 months due to suspicion of macrocephaly. Some mild enlargement in the ventricles and extra-axial fluid spaces were noted but did not really appear to be clinically significant. The maternal family was also noted to have mild familial macrocephaly.

Behavior: The family of this patient reported some aggressive behavior starting at the age of 11, including mainly self-injurious behavior (striking his head and temple, etc.) and some physical aggression towards the family. The frequency and duration significantly decreased once he reached 14 years of age (1-2 per year, lasted no more than a minute compared to 5-10 minutes before).

Sequencing studies: Exome and WGS independently identified a *de novo* c.1819G>A (p.Asp607Asn) mutation in *AHDC1*. The mutation is also absent in his four unaffected siblings. Other possible candidates are listed in Table 3.

Referral: This family consented to participation in the XGS Registry.

Case 7: is a 12-year-old female with relative microcephaly, developmental delay, tight heel cords, significant behavior problems and sleep disruption. The pregnancy was complicated by a diagnosis of Lyme disease during the second trimester. This patient was delivered by emergency C-section at 42 weeks of gestation due to breech position. She had feeding difficulty for the first six months and had gastroesophageal reflux requiring treatment in the first year. She sat at 10-11 months and walked at 14-15 months. She spoke her first word beyond 2 years of age. At present, she is using full sentences with a vocabulary of more than 200 words, but she has trouble with receptive language. The patient is of tall stature, 82% in height at 8yo.

Renal and bladder MRI at the age of 9 showed that she has a small cyst in her left kidney and she has a small and thick-walled bladder.

Neurology: Brain MRI at 3 years showed molar tooth sign. Brain MRI at 10yo showed craniofacial and brain malformations, compatible with genetic encephalopathy and Joubert syndrome-related disorder. This patient had a full-scale IQ in the range of 68 and 72.

Behavior: This patient had long-standing behavioral issues since she was a toddler. She has been on multiple medications for her behavior since 3-4yo. She has long-standing ADHD in addition to depression and anxiety and has been diagnosed with disruptive mood disorder. Her family reported worse behavior since approximately age 9 with some puberty changes. Her behavior seems to worsen on approximately a monthly cycle, but she has yet started menstruation. She was hospitalized at age 10 years due to suicidal and homicidal ideation.

Sequencing studies: This patient was initially tested by chromosomal microarray, and a deletion of 2q13 and a duplication of region Xq27.1 were identified. The Xq27.1 duplication was also found in her mother. The 2q13 deletion, which encompasses the *NPHP1* gene that is associated with autosomal recessive Joubert syndrome, appeared to be paternally inherited per exome sequencing. Exome sequencing identified a *de novo* c.2374G>C(p.Gly792Arg) mutation in *AHDC1* and was classified as a variant of uncertain significance; no additional variants in NPHP1 were identified by ES.

Referral: This family consented to participate in the XGS Registry.

Case 8: is a 10-year-old male registered in the DECIPHER with autism and global developmental delay. Dysmorphic features included esotropia, hypermetropia, microtia, brachycephaly, a broad forehead, horizontal eyebrows, a wide nasal bridge and 5th finger clinodactyly. The patient suffered from recurrent respiratory infections and had an intussusception as an infant.

Neurology: Global developmental delay, absent speech at 6 years, and autism spectrum disorder. MRI head at 11 months showed ventriculomegaly and decreased white matter. Occipitofrontal circumference measurements at 2 and 6 years showed postnatal microcephaly.

Sequencing studies: through the DDD project, a *de novo* missense variant was reported in *AHDC1* (c.4042T>C, p.Ser1348Pro) and a *de novo* hemizygous mutation was reported in *HUWE1* (c.9070G>A, p.Ala3024Thr). He is also compound heterozygous for two *NEB* variants (c.9139C>A, p.His3047Asn, paternal and c.7343G>A, p.Arg2448His, maternal). Previous ClinVar classifications of these variants are as variants of uncertain clinical significance and as LB. There are no reported features of a nemaline myopathy. They also had a normal karyotype and array-CGH (comparative genome hybridization),

Referral: Phenotype and genotype are from the DECIPHER database and a clinical notes review.

Case 9: Refer to Gumus (2020).

Case 10: is a 11-year-old female with global developmental delay, microcephaly, gait abnormalities, and speech apraxia. There has been a major concern with recurrent falls, one of which led to a fractured elbow that required surgery. She is unable to run. She wears braces on her feet. She has difficulty with sleep onset for which she takes melatonin. She has been evaluated in sleep medicine and has had a normal sleep study. An iron deficiency was identified for which she takes supplemental iron. She has deficient upper right premolars. Her ears are normally positioned and well-formed. Eyes are spaced appropriately. Eyebrows are normal. Palpebral fissures are

slightly upslanting. Irides are intact. Nose is normal. Philtrum and lips are normal. Jaw is well formed. She has a history of conductive hearing loss. Her mother also reports that she has joint and muscle pain. She has a 6-year-old sibling who is apparently normal.

There were no teratogenic exposures or illnesses during pregnancy and no neonatal problems. Development was delayed, and hearing loss has been an issue that responded to PE tubes and was secondary to numerous infections. She has no history of hospitalizations. Her immunizations are up to date. At present time, she may use approximately 10 single words but does not use word combinations. She exhibits relatively good comprehension. She is able to express her wants and needs using a tablet computer and a picture-based system. There has been no interval regression of any of her language skills.

Neurology: Brain MRI completed at 8 years identified dysgenesis of the corpus callosum and deep white matter T2 hyperintensity. She has had no seizures.

Behavior: She has behavioral concerns including tantrums. In school, she receives autism services, but it is primarily language that is her problem. She likes to socialize. She likes to communicate as well as she can, and there is evident eye contact. Her mother also explained that she was felt to have a lot of words until she was about 2 years of age and then lost the use of some of these.

Sequencing studies: Exome sequencing identified a VUS in *AHDC1* (c.4432C>T, p.P1478S). The variant was not inherited from the mother, but it is unclear if the father has the same variant. An additional heterozygous variant in *UBE3B* was detected that is known to cause Kaufman

oculocerebrofacial syndrome in an autosomal recessive fashion; hence, it is unlikely to explain the observed clinical features.

Referral: This family consented to participate in the XGS Registry.
